# Nuclear Importation of *Mariner* Transposases among Eukaryotes: Motif Requirements and Homo-Protein Interactions

**DOI:** 10.1371/journal.pone.0023693

**Published:** 2011-08-18

**Authors:** Marie-Véronique Demattei, Sabah Hedhili, Ludivine Sinzelle, Christophe Bressac, Sophie Casteret, Nathalie Moiré, Jeanne Cambefort, Xavier Thomas, Nicolas Pollet, Pascal Gantet, Yves Bigot

**Affiliations:** 1 PRC, UMR INRA-CNRS 6175, Nouzilly, France; 2 GICC, UMR CNRS 6239, UFR des Sciences et Techniques, Tours, France; 3 IRBI, UMR CNRS 6035, UFR des Sciences et Techniques, Tours, France; 4 UMR INRA 0483, UFR de Pharmacie, Tours, France; 5 Metamorphosys, CNRS UPS3201-Université d'Evry Val d'Essonne, Genavenir 3 - Genopole Campus 1, Evry, France; 6 CIRAD, UMR 1098 Développement et Amélioration des Plantes, Montpellier, France; University of Poitiers, France

## Abstract

*Mariner*-like elements (MLEs) are widespread transposable elements in animal genomes. They have been divided into at least five sub-families with differing host ranges. We investigated whether the ability of transposases encoded by *Mos1*, *Himar1* and *Mcmar1* to be actively imported into nuclei varies between host belonging to different eukaryotic taxa. Our findings demonstrate that nuclear importation could restrict the host range of some MLEs in certain eukaryotic lineages, depending on their expression level. We then focused on the nuclear localization signal (NLS) in these proteins, and showed that the first 175 N-terminal residues in the three transposases were required for nuclear importation. We found that two components are involved in the nuclear importation of the *Mos1* transposase: an SV40 NLS-like motif (position: aa 168 to 174), and a dimerization sub-domain located within the first 80 residues. Sequence analyses revealed that the dimerization moiety is conserved among MLE transposases, but the *Himar1* and *Mcmar1* transposases do not contain any conserved NLS motif. This suggests that other NLS-like motifs must intervene in these proteins. Finally, we showed that the over-expression of the *Mos1* transposase prevents its nuclear importation in HeLa cells, due to the assembly of transposase aggregates in the cytoplasm.

## Introduction

Transposable elements (TEs) are genomic DNA sequences that can move and duplicate autonomously or with the assistance of other elements within genomes. They are present in almost all the organisms in which they have been sought, and can make up a large proportion of a genome, for example, 45% of primate genomes, 38.5% of the mouse genome, 5% of euchromatin in *Drosophila melanogaster*, and in some plants, such as maize, they can account for 80% of the genome (for review [1]). TEs are divided into two main classes on the basis of their structural organization and transposition mechanism [2]. Members of the larger family of retroid agents, that includes the retroviruses, are known as Class I TEs. They use an RNA-mediated mode of transposition. In contrast, the Class II TEs, the transposons (*sensu stricto*), use a DNA-mediated mode of transposition. Two types of transposons compose Class II TEs in eukaryotes. The first belongs to two superfamilies, *Helitron* [3] and *Maverick*/*Polinton* [4]. A characteristic that differentiates them of the second type of Class II TEs is that their origin seems to be common with those of some virus families, including Geminivirus and Maviruses. The molecular modalities of their mobility remain, however, to be elucidated. The second type is composed of the cut-and-paste DNA transposons that are grouped into at least 17 superfamilies, based on sequence similarity of element-encoded transposases [5]. The *IS630-Tc1-mariner* (*ITm*) superfamily is one of them and is probably the most widespread among eukaryotes [5]. Its members are grouped into 7 families: *IS630*, ITmD34E (so-called *Tc1*-like element (TCE or TLE)), ITmD34D (so-called *mariner*-like elements (MLE)), ITmD37D (so-called *maT*), ITmD37E, ITmD39D (incorrectly named plant MLEs since their discovery [6]), and ITmD39D (so-called *Gambol*) (for review see [5]–[9]).

Among *ITm*, the MLE family is by far the most widespread among animal genomes. MLEs are transposons of 1200 to 2000 base pairs (bp) in length that contain a single intron-less gene encoding the transposase. This gene is flanked by two short inverted-terminal repeats (ITRs) of 19 to 40 bp in length [10]. The transposase allows transposition to occur *via* a DNA intermediate and a specific binding to its ends [9]. The MLE family consists of five sub-families designated *cecropia*, *elegans*/*briggsae*, *irritans*, *mauritiana* and *mellifera/capitata* [11]. The discovery of *Tvmar1* in the genome of *Trichomonas vaginalis* has suggested that there may be a sixth sub-family [12]. Based on sequence phylogeny between their element-encoded transposase, an empirical rule is that below a threshold of 45% of sequence similarity, two MLEs generally belong to two different sub-families, whereas at a threshold over 45% they are considered sibling elements belonging to a single sub-family. The prevalence of various MLE sub-families varies in different taxonomic animal groups. Members of the *elegans*/*briggsae* and *mellifera*/*capitata* sub-families have so far each been reported to occur in only one kind of host, respectively, nematodes and insects. In contrast, MLEs belonging to the *mauritiana*, *cecropia* and *irritans* sub-families have been found in at least one invertebrate and one vertebrate family. Some studies have indicated that the *irritans* sub-family consists of several lineages, which are related to the host of origin [13], [14]. Altogether, these observations suggest that some comparable and different properties, including common sequences and mechanisms of integration, among MLE transposases may have been either retained or diverged depending on functional constraints conferred by their host lineage in which they have evolved. Indeed, a part of their differences could not only involve their interactions with specific host factors that are necessary for transposition in the nucleus, but also in the upstream steps involved transposase synthesis.

Although mechanistic data is limited in current literature, it is clear that several cellular processes act upstream of MLE transposition and that these can modulate its efficiency. At least 5 criteria, arguable, are required for efficient transposition: (1) the transposase gene is transcribed in the nucleus, then (2) the transposase transcripts are processed and exported into the cytoplasm, before (3) being translated into a protein. The nascent transposase is then subjected to (4) a proper folding, then post-translational modifications, before being (5) internalized into the nucleus where it mediates transposition. One or more of these steps might be transposition limiting. For example, previous studies of the *Mos1* and *P* transposons have demonstrated that post-translational modifications could decrease transposase activity [15], [16].

Our work reported here focuses on the information contained in the sequence of MLE transposases that allow them to be imported into the nuclei. The machinery for protein nuclear importation is highly conserved in animals, fungi and plants. In general, it involves specific signals, known as nuclear localization sequences (NLSs), which are contained in the protein sequence. Though no systematic consensus NLS can be determined with absolute reliability, two categories of NLS have so far been characterized in some detail. They correspond to monopartite ([K/R]_4–6_) and bipartite ([K/R]_2_X_10–12_[K/R]_3_) motifs consisting of short stretches of basic residues, such as lysines (K) and arginines (R), and histidines (H) in some cases [17], [18]. When such NLSs are involved in nuclear import of a protein, they are generally located in a non structured segment that contains or are juxtaposed by one or several prolines, and are exposed at the protein surface. Current literature indicates that the importation of the transposase into the nuclei can be achieved in different ways, depending on the transposon family. Some transposases depend on the presence of one or several NLS in their sequence that correspond to monopartite or bipartite motif(s), as previously demonstrated for certain Ac, and *Mutator* [19], [20]. Nuclear importation can also involve non-cardinal motifs, as has been demonstrated for *BmTc1*, *Hermes* and *piggyBac* [21]–[23]. In the absence of NLS, a cargo protein can be involved, as exemplified by the *Harbinger3_DR* transposon that encodes a second *myb*-like protein required for the nuclear importation of the transposase [24].

To date, only one experimental work has been published on the nuclear importation of some *ITm* transposases [25]. The study focused exclusively on the demonstration that an active bipartite NLS is highly conserved in the sequence of TLE transposases. From the earliest work on *ITm* elements, it is clear that the bipartite NLS found in TLE transposases is not conserved in those of the six other families (see Figure 2 in [26]). Nevertheless, it was suggested that there would be a bipartite NLS in the *Mos1* transposase [25], [27] and two monopartite NLS in the *Himar1* transposase [28]. Interestingly, these motifs are not highly conserved in the sequence of sibling transposases of their own MLE sub-families and are absent in those of the other sub-families [9], [26].

Here, we investigated the ability of three different MLE transposases to enter the nucleus in eukaryotic cells. These active MLE transposases (MOS1, HIMAR1 and MCMAR1; [29]–[31]) are encoded by natural MLEs that belong to three different sub-families: *Mos1* for *mauritiana*, *Himar1* for *irritans* and *Mcmar1* for *elegans*, respectively. Their nuclear import abilities have been assayed in four different cell types that vary in the relative occurrence of MLEs in their genome: a plant cell, that belong to a taxon in which genomes are devoid of MLEs; an insect cell, a taxon in which members of all MLE sub-families occur, except *elegans*/*briggsae*; and amphibian and mammal cells, taxa in which members of the *mauritiana*, *irritans*, and *cecropia* sub-families occur. Our findings show that MOS1, HIMAR1, and MCMAR1 differ in their abilities to enter the nucleus of all four cells. The nuclear importation of these proteins is modulated by at least two sequences located in their N-terminal region: a nuclear dimerization domain and a nuclear localization signal (NLS).

## Results

### *In silico* search for NLSs in MLE transposases

**Figure 1:**
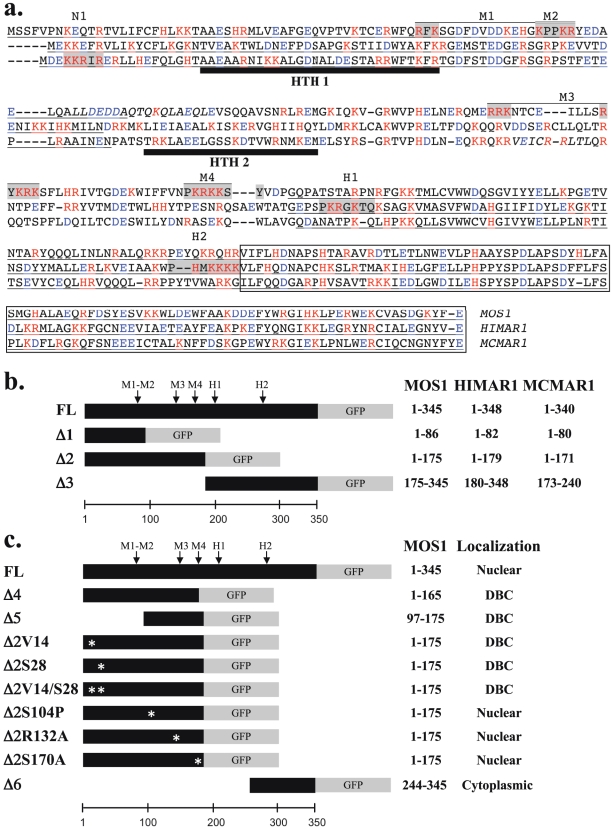
Sequence features of MOS1, HIMAR1 and MCMAR1. (a) Alignment of the amino acid sequences of three MLE transposases. Basic residues, lysine (K), arginine (R) and histidine (H) are typed in red. Acidic residues, aspartate (D) and glutamate (E) are typed in blue. The C-terminal region with an acidic pI is boxed. Motifs corresponding to potential NLS in MLE sequences are boxed in grey and indicated above the sequence. The four putative monopartite and bipartite NLSs found *in silico* in MOS1 are referred to as M1, M2, M3 and M4. M1 is a putative bipartite NLS (RFK---KPPKR), whereas M2 is monopartite (KPPKR). Monopartite NLSs found in the HIMAR1 sequence are referred to as H1 and H2. HTH1 and HTH2 are located in the N-terminal domain. The regions corresponding to the Δ1 and the Δ3 segments are underlined. (b) Schematic representation of the transposase-GFP fusions used to explore the subcellular localization of MOS1, HIMAR1 and MCMAR1 in onion epidermal cells, drosophila cells, human cells and *X. tropicalis* cells. The amino acid at borders of each transposase segments are indicated in the right margin. (c) Schematic representation of the different truncated and mutant versions of MOS1-GFP fusion used in the study. The amino acid length of each transposase segment as well as the fluorescence results are indicated in the right panel. Mutated residues in each fusion are located with an “Ψ”.

In an attempt to locate putative monopartite and bipartite NLSs, we first conducted an *in-silico* analysis using the amino acid sequence of three active *mariner* transposases: MOS1, HIMAR1 and MCMAR1 belonging to the *mauritiana*, *irritans* and *elegans* sub-families, respectively. All the sequences of MLE transposases so far described are rich in basic residues (Figure 1, red letters). As a consequence, they have a predicted high IEP (isoelectric point; Table 1). Sequence analysis of the three transposases revealed that basic residues are not homogenously distributed, and two regions with very different IEP values can be distinguished (Figure 1, Table 1). The N-terminal regions spanning from residues 1-237/246 were richer in basic residues than the region consisting of the last 101–102 C-terminal residues. Taking into account the distribution of the basic residues in MLE transposases, motifs characteristic of NLSs, consisting of H, K and R located in the N-terminal region can be expected to be present. Moreover, sequence comparisons revealed that there was no stretch of basic residues, conserved in a sequence and location that would make it possible to consider them as common monopartite or bipartite NLSs shared by MOS1, HIMAR1 and MCMAR1. This therefore supports the hypothesis that no cardinal conserved monopartite or bipartite NLS was present in these three MLE transposases, and that each of them uses different NLSs. Searches for cardinal and degenerated NLSs were monitored using PredictNLS at http://cubic.bioc.columbia.edu/predictNLS/ or PSORTII at http://psort.ims.u-tokyo.ac.jp/form2.html. We detected 2 monopartite and 2 bipartite putative NLSs in MOS1 (Figure 1a, grey boxed letters, designated M2 and M4, and M1 and M3, respectively), 2 monopartite NLSs in HIMAR1 (Figure 1a, grey boxed letters, designated H1 and H2), and 1 in MCMAR1 (Figure 1a, grey boxed letters, designated N1). All these NLSs were degenerate, apart from M3, which was previously proposed to be an active bipartite NLS in MOS1 [25], [27] and M4, which was very similar to that of the SV40 T antigen. Interestingly, taking into account the structure of MOS1 [32] and the alignment of three sequences of transposase, we observed that M1, M3, H2 and N1 are included in with α-helix or β-sheet folds, what strongly impairs that they are functional NLS.

**Table 1:** IEP of the MLE transposases.

Transposase	pI
*Mos1*	
Full-length	9.07
N-terminal region: 1-243	9.73
C-terminal region: 244-345	5.26
*Himar1*	
Full-length	9.07
N-terminal region: 1-246	9.45
C-terminal region: 247-348	6.10
*Mcmar1*	
Full-length	8.84
N-terminal region: 1-237	9.51
C-terminal region: 238-340	5.39

### Nuclear importation of the MLE transposases in animal and plant cells

**Figure 2:**
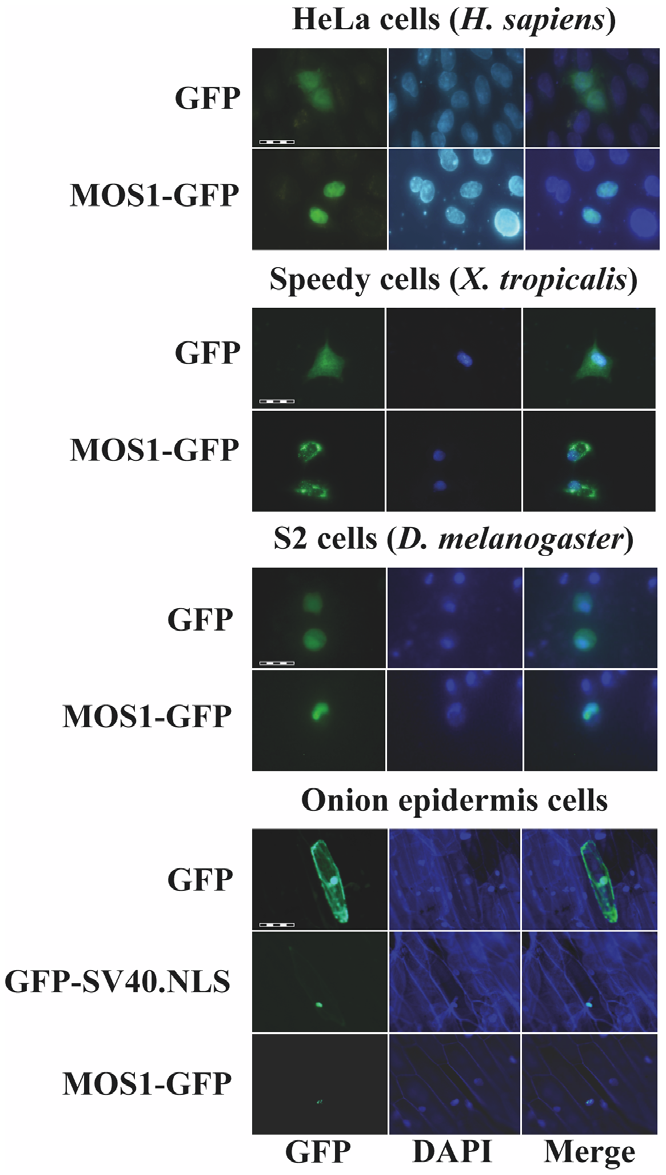
Localization of MOS1 in host cells. The GFP fluorescence patterns are analyzed in human HeLa cells, amphibian cells, insect cells, and onion epidermal cells transfected with plasmids expressing only GFP or a MOS1-FL GFP fusion. The left panels show GFP fluorescence, the middle panels show the nuclear genomic DNA staining by DAPI, the right panels correspond to merge pictures. For onion epidermal cells, the pattern of a GFP-SV40.NLS fusion was also verified since the localization of the MOS1-FL GFP was not homogenous in the nuclei. The scale bars correspond to 100 µm in HeLa cells, 200 µm in amphibian cells, 50 µm in insect cells, and 200 µm in plant cells.

The ability of MOS1, HIMAR1 and MCMAR1 to be actively concentrated in the nuclei of animal and plant cells was first investigated by transiently transfecting expression plasmids encoding the full-length (FL) transposases fused at their C-terminal end with GFP (green fluorescent protein; Figure 1b,c). The subcellular localization of the three fluorescent protein fusions was investigated in mammalian cells (HeLa), amphibian cells (*Xenopus tropicalis,* speedy cell line), insect cells (Drosophila S2 cells) and in plant cells (onion epidermis). As a control, transfections with plasmids expressing GFP were also performed to distinguish the nuclear and cytoplasmic distribution of the fluorescence in each cell type (Figure 2). The MOS1 FL-GFP was found to be located within the nucleus in the onion epidermis, insect and HeLa cells, but exclusively cytoplasmic in *X. tropicalis* cells (Figure 2), in which it had assembled into small cytoplasmic aggregates. Further investigations done by injecting mRNA encoding MOS1 FL-GFP in *X. laevis* embryos however showed that MOS1 was able to localize into nuclei when it was present at a low concentration in amphibian cells (Supporting Information S1 and unpublished data, LS and YB). The HIMAR1 FL-GFP chimera was the only recombinant protein that was found to be specifically located in the nucleus of all four cell types (Supporting Information S2a). The MCMAR1 FL-GFP fusion was only found in the nucleus of HeLa and insect cells (Supporting Information S2b). Interestingly, MCMAR1 FL-GFP was exclusively cytoplasmic in the other two cell lines. These results indicated that the three MLE transposases contain sequence information required for their nuclear importation. The HIMAR1 GFP fusion protein was located within the nucleus in all the cell lines tested, whereas the presence of MOS1 and MCMAR1 fusions in the nucleus depended on the host cell type.

### Experimental localization of the transposase region containing active NLS

Proteins with a molecular weight of less than 40–60 kDa are thought to be able to enter the nucleus by simple passive diffusion [16]. As a result, it is generally recommended that marker proteins of more than 60 kDa, such as the GFP-GUS fusion protein, (79.6 kDa [10], [22]) should be used to study nuclear importation. However, proteins actively imported into the nucleus can also contain a nuclear export signal (NES [33], [34]) or a cytoplasmic retention motif, which acts as an antagonist of the NLS, depending on the physiologic state of the cell, the cell type and/or post-translational modifications [27], [28]. To detect antagonism due to the presence of NES, or cytoplasmic retention motifs, or other retention mechanisms, we assayed constructs containing fusions of GFP with modified versions of each three transposases. They were made with three truncated segments Δ1, Δ2 and Δ3 that were fused at their C-terminal ends with GFP (the sequence location of each segment is given in Figure 1b). The subcellular localizations of the GFP fusions are summarized in Table 2. Regardless of the host cell, no active nuclear concentration of the GFP fluorescence was observed with the three Δ1-GFP fusions (about 35 kDa each) or the three Δ3-GFP fusions (about 43 kDa each). These results showed that some putative NLSs identified within the HIMAR1 sequence (H1 and H2, in Figure 1a), within the MOS1 sequence (M1 and M2 in Figure 1a) and within the MCMAR1 sequence (N1 in Figure 1a) were not functional. Results obtained with the MOS1, HIMAR1 and MCMAR1 Δ2-GFP fusions (about 46 kDa each) in HeLa, Drosophila S2, and onion epidermis cells were similar to those observed with the FL-GFP fusions. GFP fluorescence was observed only in the nuclei. These findings indicate that the region spanning from approximately amino acids 80 to 175 contains information required for the nuclear importation in the three transposases. MOS1 Δ2-GFP was also located in the nucleus in amphibian cells. Fluorescence due to the HIMAR1 Δ2-GFP and MCMAR1 Δ2-GFP fusions diffused into both cellular compartments in amphibian cells. This suggested either that the NLS information contained between amino acids 1 to 175 in the HIMAR1 and MCMAR1 sequences was not powerful enough to allow a high degree of localization into the nuclei of *X. tropicalis* cells, or that a steric hindrance by GFP prevents the recognition of the NLS information by the proteins involved in the nuclear import of *X. tropicalis* cells.

**Table 2:** Subcellular localization of transposase/GFP fusions.

Transposase-GFP fusions	Human cells (HeLa)		Amphibian cells *X. tropicalis* (Speedy)		Insect cells (S2)		Plant cells Onion epidermis	
	Nucleus	Cytoplasm	Nucleus	Cytoplasm	Nucleus	Cytoplasm	Nucleus	Cytoplasm
MOS1								
FL-GFP	+	-	-	+	+	-	+	-
Δ1-GFP	DBC		DBC		DBC		DBC	
Δ2-GFP	+	-	+	-	+	-	+	-
Δ3-GFP	DBC		DBC		DBC		DBC	
HIMAR1								
FL-GFP	+	-	+	-	+	-	+	-
Δ1-GFP	DBC		DBC		DBC		DBC	
Δ2-GFP	+	-	DBC		+	-	+	-
Δ3-GFP	DBC		DBC		DBC		DBC	
MCMAR1								
FL-GFP	+	-	-	+	+	-	-	+
Δ1-GFP	DBC		DBC		DBC		DBC	
Δ2-GFP	+	-	DBC		+	-	+	-
Δ3-GFP	DBC		DBC		DBC		DBC	

+, presence; -, absence; DBC  =  Diffused into both compartments.

**Figure 3:**
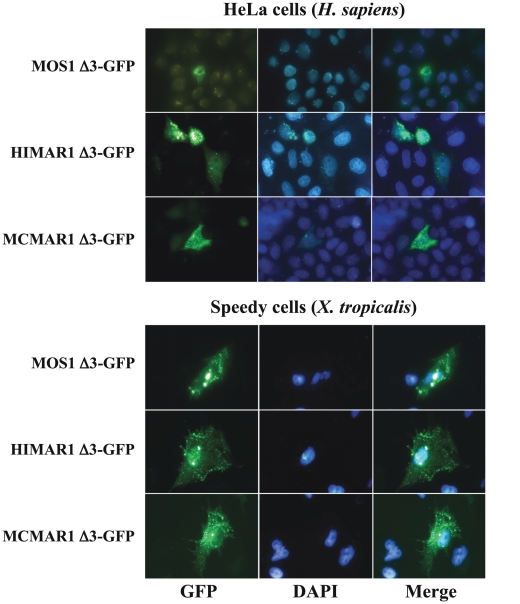
Localization of transposase Δ3 GFP fusions in host cells. The GFP fluorescence patterns are analyzed in human HeLa cells and amphibian cells transfected with plasmids expressing only MOS1-Δ3 GFP, HIMAR1-Δ3 GFP or MCMAR1-Δ3 GFP fusions. The left panels show GFP fluorescence, the middle panels show the nuclear genomic DNA stained by DAPI, the right panels correspond to merge pictures.

Some of our data indicated that information contained in the C-terminal region of MOS1 and MCMAR1 was able to antagonize the localization of the transposase into the nuclei. This was supported first by the observation that the MOS1 Δ2-GFP fusion was localized in the nuclei of the *X. tropicalis* cells, whereas the MOS FL-GFP fusion was not. Similarly, MCMAR1 Δ2-GFP was localized in the nuclei of the onion epidermis cells, whereas the MCMAR1 FL-GFP fusion was not. The existence of an antagonist sequence in the C-terminal region of the MLE transposases was also supported by the fact that the assembly of fluorescent cytoplasmic aggregates could be observed in cells that express the Δ3-GFP fusions. Our observations indicate that the presence of aggregates depends considerably on the cell type, the fusion sequence and the expression level. We observed that numerous small fluorescent aggregates were present in all *X. tropicalis* cells with the three Δ3-GFP fusions, thus most likely preventing their diffusion and/or import into the nucleus (Figure 3). These aggregates were larger and less abundant in HeLa cells, and only present when the rate of expression of Δ3-GFP fusions was high (Figure 3). In Drosophila S2 and onion epidermis cells, such aggregates occurred only in the cells expressing the MCMAR1 Δ3-GFP fusion (Supporting Information S3). Previous studies [32], [35] have indicated that these observations did not result from the presence of an NES or a cytoplasmic retention motif in the C-terminal domain of MOS1, HIMAR1 and MCMAR1, but from protein-protein interactions. Indeed, two domains located within the first 50 N-terminal and the last 50 C-terminal residues of the MLE transposases were found to be essential for the assembly of a transposase dimer that is necessary for transposition, and that of an inactive transposase dimer or oligomer [35], respectively. The balance between the two pathways of transposase-transposase interaction has been proposed to be concentration dependent [35]. Our data indicate that oligomers are assembled in aggregates in the absence of the first 50 N-terminal residues when the Δ3-GFP fusions that contain the C-terminal oligomerization domain are over-expressed in cells.

### Requirements for nuclear import in the N-terminal region

**Figure 4:**
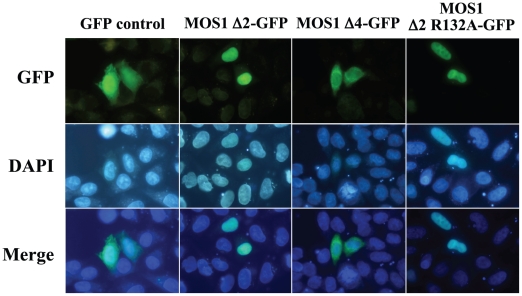
Comparisons of fluorescence patterns between MOS1 Δ2-GFP and two variants. The GFP fluorescence patterns are analyzed in HeLa cells transfected with plasmids expressing only GFP or MOS1 Δ2-GFP as controls, MOS1 Δ4-GFP or MOS1 Δ2 R132A-GFP variants. The top panels show GFP fluorescence, the middle panels show the nuclear genomic DNA staining by DAPI, the bottom panels correspond to merge pictures. The observation of aggregates was already reported in the literature with the transposon protein MURB (about 26 kDa; [20]). This accessory protein is encoded by the plant transposon *MuDR* and assembles aggregates in the cytoplasm when it has an important expression rate.

In order to identify the motifs contained in the N-terminal regions of the MLE transposases that are able to elicit efficient import into nuclei, we focused our efforts on the localization of MOS1 in HeLa cells. At first, plasmid constructs were made to verify the functionality of the four potential monopartite or bipartite NLSs (M1 to M4) found in MOS1 by fusing them at their C-terminus with GFP (Figure 1a). Results revealed that none of these motifs was able to actively concentrate the GFP-fluorescent signal in the nucleus. For M4, this finding was unexpected, because it has a sequence very similar to that of the SV40 NLS (PKRKKSY *versus* PKKKRKV). Furthermore, this sequence matches the essential requirements for a monopartite NLS, i.e. it has a K at the second position and basic residues at the third and fifth positions [17]. Moreover, it is located in a non structured segment and contains a proline at the first position [17], [32]. This was confirmed using M4-GFP fusions with a small peptide linker inserted between the moieties, as recommended [36]. However, these results could not be relied upon because we obtained similar results in HeLa cells using two different constructs expressing an SV40-NLS-GFP or an SV40-NLS-linker-GFP. An alternative approach was therefore developed to determine whether M4 was essential for the nuclear importation of MOS1. A plasmid expressing the first 165 residues of the transposase fused to GFP (MOS1 Δ4-GFP construct, Figure 1c) was made. This truncated version of MOS1 transposase contained M1, M2 and M3, but not M4. Transfection results in HeLa cells revealed that the GFP-fluorescent signal did not concentrate in the nuclei in the absence of M4 (data not shown). This indicated that M4 was an essential NLS motif for the active transportation of MOS1 into the nuclei. Similarly, to find out whether the MOS1 region spanning from residues 97 to 175 contained enough information to actively concentrate the GFP-fluorescent signal in HeLa cell nuclei, a plasmid construct was made to fuse it at its C-terminal with the GFP (MOS1 Δ5-GFP; Figure 1c). Our results showed that the GFP-signal was not concentrated in the nuclei (Figure 4). This last finding was unexpected and led us to conclude that information contained within the first 96 amino acid residues must be required for the active transportation of MOS1 into the nucleus.

The first 50 N-terminal residues in MOS1 contain a homeodomain-like structure composed of three α-helices [30], [32]. The first of these is involved in transposase-transposase interactions. MOS1 dimerization was therefore impaired in three Δ2-GFP mutants by making a single or double proline substitution at positions V14 and S28 (Figure 1c). The analysis of the plasmid transfected in HeLa cells revealed that the three mutants were unable to concentrate the GFP-fluorescent signal in the nuclei (data not shown). These findings demonstrated that a functional dimerization domain is required for the nuclear importation of MOS1.

### MOS1 nuclear importation is not affected by certain point mutations

Based on published results of MOS1 mutants [15], [27], [37], we examined the effects of three amino acid substitutions on the functionality of the nuclear importation of MOS1 (Figure 1c). The first corresponded to substitution S104P, which is located within the helix-turn-helix (HTH) of the second homeodomain contained in the N-terminal region of MOS1 (Figure 1a, HTH2) [30]. Since prolines are secondary structure breakers, this substitution had a dramatic effect on the HTH structure. It was also found that the S104P mutant had modified MOS1-MOS1 interactions [37], and was consequently unable to bind to its ITRs [15]. The second mutation was the R132A substitution that had previously been proposed to be located into a bipartite NLS [25], [27] and to have an impaired ability to be actively imported into nuclei [38]. The third was substitution S170A, which is located at the NLS4 end. S170 is a highly phosphorylated residue in MOS1 extracts purified from baculoviral production in Sf21 insect cells (personal data, YB). Its substitution by an alanine produced a 50-fold decrease in the ability of MOS1 to mediate transposition [15]. Each of these mutations was introduced into a Δ2-GFP fusion, and their GFP profiles were analyzed. The results revealed that all three mutants were still efficiently translocated to the nuclei (Figure 4). These findings therefore indicated that i) the second homeodomain located at positions 80 to 115 in the N-terminal region [15] did not interfere with nuclear importation, ii) residue R132 was not involved in the nuclear importation of MOS1, and iii) the absence of phosphorylation at position 170 did not impair the nuclear importation of MOS1.

### Nuclear importation of a humanized MOS1 variant in HeLa cells

**Figure 5:**
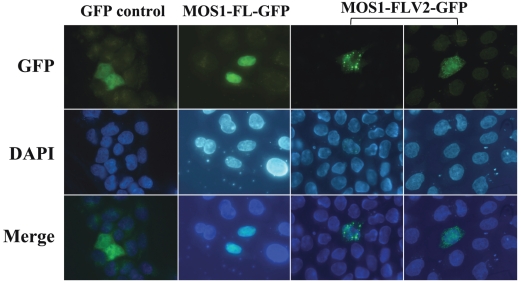
Localization of a highly expressed MOS1 in host cells. The GFP fluorescence patterns are analyzed in HeLa cells transfected with plasmids expressing only GFP or MOS1-FL GFP as controls, and MOS1-FLV2-GFP. In both MOS1-FLV2-GFP patterns, exposure times were 5-fold and 10-fold shorter than those obtained with GFP or MOS1-FL GFP. The top panels show GFP fluorescence, the middle panels show the nuclear genomic DNA staining by DAPI, and the bottom panels correspond to merge pictures.

Regardless of cell type, the expression rate of a GFP fusion is a parameter that is extremely difficult to monitor in the context of transient transfection, since it depends on the plasmid transfection efficiency and the fate of the plasmid in each cell. For example, under our experimental conditions, the transfection of the pCS2-GFP plasmid in HeLa cells leads to GFP expression that varied over nearly four orders of magnitude (Supporting Information S4). To clarify the situation, we investigated the impact of the MOS1 over-expression on its ability to be actively concentrated in the nuclei. Since the gene encoding MOS1 was of drosophilan origin, we constructed a new version, designated MOS1V2, by optimizing codon usage, removing the putative intrastrand dyadic structures, and cloning it in a pCS2-GFP plasmid optimized for expression in HeLa cells by adding UTRs (Supporting Information S5). We found that the expression of this optimized plasmid construct increased MOS1 production by about 15-fold higher than that of its wild-type version (Supporting Information S5). The expression of this optimized plasmid was followed by fluorescent microscopy. The results indicated that the MOS1V2-GFP was not localized in the nuclei in most of the cells. They also showed that the GFP-fluorescent signal was concentrated in aggregates with granular or fibrous shapes that are only present within the cytoplasm (Figure 5). These observations suggested that MOS1V2-GFP assembled in large oligomers when it was present in the cytoplasm at a higher concentration than that resulting from the expression of the non-optimized expression plasmid. Its restricted presence in the cytoplasm and absence from the nuclei indicated that the assembly of the aggregates probably occurs in the vicinity of the MOS1 synthesis sites and/or along the cytoskeleton fibers.

Nevertheless, our observations raised questions about the biological significance of these aggregates since they might result from an artifactual property of the MOS1V2-GFP fusion that the natural MOS1 would not have. We have never been able to obtain polyclonal antibodies directed against MOS1 in spite of significant efforts developed during several years with several vaccination strategies and animal species. To circumvent this problem, we have therefore developed several approaches to verify whether or not an active MOS1 was able to aggregate.

First, we made vector V5-MOS1V2 by fusing a tag (the V5-antigen: MGKPIPNPLLGLDST) to the N-terminal end of MOS1V2. V5-MOS1V2 is therefore a fusion quite different from MOS1V2-GFP and similar to the natural MOS1. We also verified that the V5-MOS1V2 was a functional equivalent of natural MOS1 using transposition assays monitored in bacteria [39]. A pCS2 plasmid expressing V5-MOS1V2 was used to transfect HeLa cells that were then incubated 24 hours at 37°C, fixed, and then incubated with a mouse anti-V5 monoclonal antibody conjugated with FITC (Invitrogen). The cellular location of V5-MOS1V2 was verified by epifluorescent microscopy. Under these experimental conditions, we obtained results similar to those obtained with MOS1V2-GFP (Figure 5). This therefore confirmed that V5-MOS1V2 assembled in aggregates when it was over-expressed in HeLa cells, supporting our hypothesis that MOS1 aggregates when in high concentration in the cytoplasm.

Based on our previous observations that the only GFP fusions able to assemble in aggregates contained the C-terminal moiety of MOS1, we made terminal deletions in the segment encoding Δ3-MOS1V2 in order to determine a minimal region responsible of this property. We found that the Δ6-MOS1V2-GFP fusion (Figure 1c) contained the minimal region of MOS1 that had an elevated propensity to assemble in aggregates when it was over-expressed in HeLa cells. Our results however did not allow us to determine whether the assembly in aggregates occurred when the Δ6-MOS1V2 is properly folded or misfolded.

**Figure 6:**
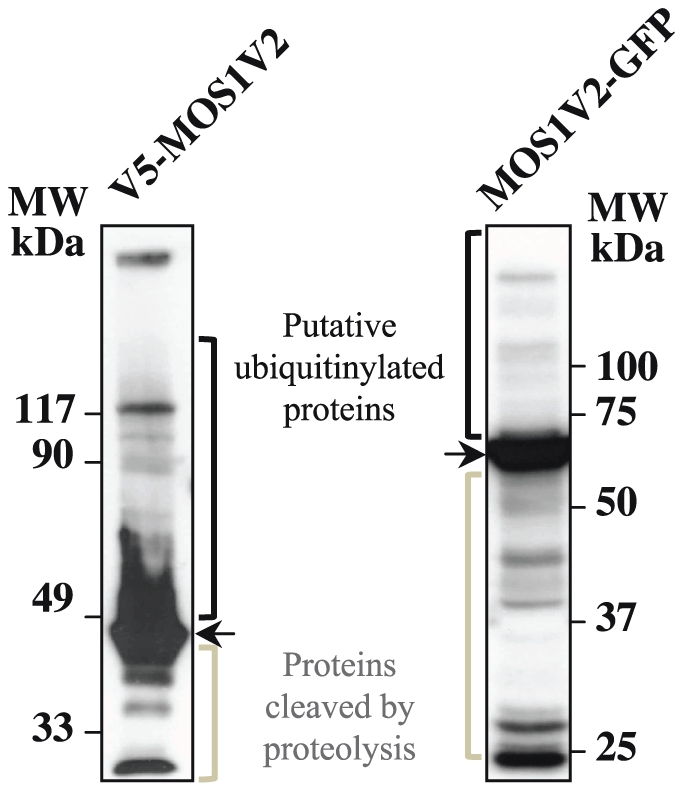
Molecular weight patterns of over-expressed MOS1 fusion in HeLa cells. Proteins extracted from HeLa cells over-expressing V5-MOS1 or MOS1V2-GFP were analyzed by immunoblotting after separation by polyacrylamide gel electrophoresis. V5-MOS1 and MOS1V2-GFP were repectively revealed by first hybridizing a mouse anti-V5 monoclonal antibody or a rabbit polyclonal anti-GFP a mouse a rabbit polyclonal anti-GFP. The filters were then incubated with horseradish peroxidase-conjugated anti-mouse IgG or anti-rabbit IgG, followed by development using enhanced chemiluminescence.

In this regard, when a protein is misfolded, it may be refolded by chaperones or tagged with lysine-linked polyubiquitin chains for degradation by the proteasome [40], [41]. When the chaperone and proteasome systems fail or are overwhelmed by over-production of proteins, misfolded proteins form oligomers and aggregates. In order to avoid cytotoxicity, polyubiquinylated aggregates are actively processed by transport systems of the cytoskeleton to form aggresomes that are then eliminated by autophagy. Whatever the pathway, a common marker of misfolded proteins is their polyubiquitinylation. Proteins subjected to polyubiquitinylation can be visualized after polyacrylamide gel electrophoresis and immunoblotting, and are revealed as a smear or in a ladder pattern with molecular masses greater than the native protein [40], [41]. Such analyses were therefore performed with proteins extracted from HeLa cells over-expressing V5-MOS1V2 and MOS1V2-GFP. Results (Figure 6) indicated that both proteins were polyubiquitynilated and partly degraded by proteolysis.

In conclusion, our results obtained with MOS1V2 support the hypothesis that a sequence is present within the last 100 residues of the MOS1 C-terminal region and it is necessary and sufficient for the assembly in aggregates when at least this region of MOS1 is misfolded following its over-expression.

## Discussion

Our results provide new information about MLEs concerning i) the ability of their transposase to be imported into nuclei among eukaryotes, ii) the motif requirements by the transposase sequence for active importation into the nucleus, and iii) the impact of transposase over-expression on their ability to be actively imported into the nucleus.

The results obtained with MCMAR1 support the hypothesis that the host nuclear importation machinery might be a barrier that has restricted the spread of some MLE sub-families in the genomes of certain eukaryotic lineages. From this standpoint, the presence of MLEs that belong to the *elegans* sub-family would be expected to be restricted to invertebrate and mammal species. The absence of *Mcmar1* relatives in vertebrate and plant species indicates that other biological factors also contribute to the delineation of their host range. However, our results indicated that host range restrictions are not due to the absence of sequence information required for nuclear importation. Indeed, the MCMAR1 Δ2-GFP variant is actively imported into the nuclei of plant cells, which indicates that other sequence or structural information distributed throughout its entire sequence allow the full-length MCMAR1 to be concentrated in the cytoplasm of plant cells. In spite of our efforts using mutagenesis, we have so far not succeeded in identifying a residue, a KDEL-like retention motif or an NES motif [33], [34] in the MCMAR1 sequence that could explain its cytoplasmic localization in amphibian and plant cells. The above properties of the full-length MCMAR1 may be due to its concentration-dependent oligomerization properties, as shown here for MOS1.

Our results obtained with MOS1 suggest that members of the *mauriatana* sub-family might not be restricted by their ability to locate into the nucleus, but its relative activity and mobility, at least in amphibian cells, may be cell-type dependent or inadvertently, its potential to assemble as aggregates in the cytoplasm if over-expressed. Regardless, MOS1 and HIMAR1 have been demonstrated to be actively imported into the nuclei of most cells. This is consistent with the numerous studies that have shown that *Mos1* and *Himar1* have a ubiquitous ability to integrate into the genome of animal species. Here, we show that MOS1 and HIMAR1 are efficiently imported to the nucleus of onion epidermis cells (a monocot plant species). Although we did not perform extensive assays in this plant, we observed that the nuclear import of MOS1 also occurred in other monocot species, such as *Oryza sativa*, and in dicot species such as *Catharanthus roseus* and *Nicotiana tabacum* [38].

The active importation of a transposase into nuclei is an essential step for *in vivo* transposition. We have demonstrated that the NLS information is contained within the first 175 N-terminal residues of the MLE transposases. Since we did not find any conserved monopartite or bipartite motif present in the HIMAR1 and MCMAR1 sequences, we focused our efforts on MOS1. We demonstrated that a monopartite motif (designated above NLS M4) has a sequence very similar to that of the SV40 NLS, and that it is the core motif in the nuclear importation of MOS1. The presence of such an NLS also indicated that MOS1 is probably imported into nuclei via the importin pathway. However, our results also indicated that the accessibility of NLS M4 in the MOS1 protein is an essential parameter of its functionality. We first observed that a protein resulting from the N-terminal fusion of NLS4 to GFP was unable to trigger the nuclear importation of the fusion. A similar problem has been reported for SV40 NLS-GFP fusions [36], and it has been shown that in this case that steric hindrance due to the proximity of the N-terminal GFP region hampered access of the NLS to the host nuclear importation machinery. Here, we have shown that NLS M4 has to be present in a transposase dimer for it to be functional. In the light of the recently published MOS1 structure [32], we observe that NLS M4 is located in a clamp loop within a non-structured protein segment that is flanked by two short β-strands (β2 and β3). Since this clamp loop is one of the two main intersubunit interfaces in MOS1 dimers, our findings suggest that NLS M4 is not accessible in the MOS1 monomer, but becomes accessible in its dimeric form, following structural modifications of the clamp loop. A similar nuclear importation system has previously been shown for STAT1 [42]. The *in silico* modelling of the HIMAR1 and MCMAR1 structures indicated that the same structural elements are probably responsible for their dimerization. However, sequence analyses revealed that these two transposases do not contain a cardinal monopartite or bipartite NLS in their sequence between residue 80 and 175. Further investigations are also necessary to determine whether the nuclear importation of HIMAR1 and MCMAR1 also depends on their dimerization.

The *in vivo* control of transposition due to overproduction inhibition (OPI) of MOS1 has been proposed as an MLE mechanism that self-regulates transposition *in vivo* [43]. *In vitro* investigations have shown the existence of a transposase concentration effect on the transposition efficiency for MOS1 and HIMAR1 [15], [44], [45]. It has been demonstrated that above a certain threshold transposase concentration inhibits transposition (10 nM for HIMAR1, and 300 nM for MOS1). However, so far there is no supporting evidence indicating whether this inhibition results from saturation of the chromosomal transposase binding sites by the transposase [46], and/or from the assembly of transposase aggregates that inactivate the transposase by conferring a configuration different from that of functional transposase dimers involved in the transposition complex [43]. In the light of these previous works and our data, two issues must be raised about our current understanding of OPI. The first concerns its biological significance, the second is related to its functioning.

OPI, for which perhaps a more appropriate denomination could be “inhibition by transposase over-concentration” (ITOC), is a phenomenon that was revealed under artificial conditions. Indeed, OPI has been observed *in vivo* in some transgenic animals [43], or in transiently transfected cells [47], [48], under conditions where the transposase over-expression is induced by moderate or strong promoter, not by the native promoter that is in general considered as weak. Similarly, there are no available data supporting whether the transposase concentrations used to reveal OPI *in vitro* are encountered *in vivo*, even under some exceptional circumstances, such as those induced by strong cellular stresses. To date, OPI is therefore a phenomenon that is relevant to several eukaryotic transposons [43]–[48], especially when they are used as vectors for gene delivery purposes. Regardless, a hypothesis that OPI could be a natural mechanism that inhibits transposition cannot be discarded, as differential expression, including elevated expression levels from native promoters, could be dependent on epigenetic factors and transient physiological changes that occur in host cells.

At present, it is not known whether OPI occurs in the nucleus, in the cytoplasm, or in both cellular compartments. Our results indicate that when MOS1 is over-concentrated in the cytoplasm of HeLa cells, it assembles in aggregates. To date, unsuccessful attempts to produce fluorescent labelling of the mitochondria or actin fibers has prevented us from determining exactly which molecular network these aggregates bind to. Although we observed this only in cells with a very intense GFP-fluorescent signal, such aggregates also occur in amphibian, insect and plant cells (Figure 3 and Supporting Information S2). Similar observations were also carried out under similar conditions of elevated GFP expression with HIMAR1 and MCMAR1, and some of their Δ3-GFP variants. Our data might therefore be the first evidence indicating that OPI occurs in the cytoplasm, where the MLE transposase may first form aggregates, and then bind to the cytoskeleton.

Aggregation of proteins that are over-expressed or accumulate in cells are linked to many diseases in human, including ageing-related neurodegeneration and systemic amyloidosis [40], [41]; [49]–[51]. In general, and especially if proteins are toxic, cells avoid accumulating protein aggregates by mechanisms including the suppression of aggregate formation by molecular chaperones, the degradation of misfolded proteins by proteasomes or the autophagy. Once formed, aggregates tend to be refractory to proteolysis and to accumulate in inclusion bodies. This accumulation has been assumed to be a diffusion-limited process. Recent studies demonstrated that aggregated proteins are specifically delivered to inclusion bodies by dynein dependent retrograde transport on microtubules in animal cells. This microtubule dependent inclusion body is called an aggresome.

Here, our results support our view that MLE transposases use a diffusion-limited process to regulate their presence in nuclei, a mechanism that is not unique to these proteins as intracellular localization of host proteins are similarly regulated. From a biological standpoint, and whether or not OPI occurs under natural conditions, it is not surprising that *in vivo* inhibition of transposition can be regulated by transposase sequestration in the cytoplasm when it is overexpressed. Indeed, cytoplasmic sequestration of such proteins is probably a cellular mechanism to protect the genome against the genotoxic consequences related to the non-specific nuclease activities of MLE transposases [35]. This also raises questions about the fate of these aggregates in the cell. They could be actively directed toward the proteasomes or be diluted over cell divisions until the transposase concentration decreases. Regardless, further studies are required to resolve the fate of the aggregates in the cells observed in our studies. For future investigations, it is likely that the *Hsmar1* MLE system [52] could be utilized as a model system to study the parameters that influence and regulate the equilibrium between transposition efficiency, transposase expression and assembly of transposase aggregates. Indeed, the three transposases used here have a transposition activity in mammalian cells that is inhibited by amino acid phosphorylation for *Mos1* [15], low for *Himar1* [29], and so far has not been shown for *Mcmar1* [31].

## Materials and Methods

### Biological materials

Onions were obtained from a local organic producer in order to avoid the possibility that the epidermis had been killed by post-harvesting X-ray treatment. The *Xenopus tropicalis* cell line used in this study (speedy cell line, unpublished data) is a secondary lineage derived from a primary lineage established from a *X. tropicalis* limb (known as 91.1.F1, kind gift of HY. Hwang). Human HeLa cells were obtained from ATCC, and *Drosophila* S2 cells from Invitrogen (Carlsbad, USA). The ORF encoding the HIMAR1 was kindly provided by David Lampe (University Duquesne, USA).

### Site-directed mutagenesis

*Mos1* transposase mutants were obtained with the Quickchange® Site-directed mutagenesis kit (Stratagene) according to the manufacturer's instructions. The template used was the gene of *Mos1* transposase cloned into the pGEM-T plasmid (Promega, Charbonnières, France). Briefly, 25 ng of template were amplified using complementary primers harboring the mutation to be introduced for 16 cycles (95°C 30 sec, 55°C 1 min, and 68°C 8 min 30 sec). The template was then degraded by *Dpn*I (3U) treatment for 1 h at 37°C, and the PCR product was transformed into XL1-blue competent cells. Mutations were verified by sequencing (MWG biotech, Germany). After sequencing, MOS1 mutant genes were subcloned in pCS2-GFP plasmids.

### Plasmid constructs

The GFP cassette originated from pCAMBIA-1302 (Acc. N° AF134298) and was fused at the C-terminal ends of the various transposase fragments. For the plant assays, the backbone expression vector was constructed from a pEMBL18 plasmid (Roche SA), in which a 1930-bp fragment of *Sph*I pCAMBIA-1302 comprising a CaMV 35S promoter, a multicloning site (*Nco*I, *Bgl*II, *Spe*I), the gene encoding the GFP and a poly-A signal from the agrobacterial nopaline synthase gene, had been cloned. This construct was designated pEMBL-GFP. The transposase encoding fragments were obtained by PCR using primers designed to contain *Nco*I and *Spe*I restriction sites, respectively, at the 5′ and 3′ ends of amplified fragments (Supporting Information S6). PCR products were cloned in pGEM-T Easy (Promega), checked by sequencing, and then cloned in frame with the GFP open reading frame (ORF) at *Nco*I and *Spe*I sites in pEMBL-GFP. The oligonucleotides encoding the four potential NLS of MOS1 were similarly cloned in pEMBL-GFP (Supporting Information S5). The accuracy of the fused ORF in each construct was also confirmed by sequencing using the primer GFPrev (5′-TGCCCATTAACATCACCATC-3′), that annealed on the minus strand of the GFP ORF, 68 to 48 bp downstream from the *Spe*I site.

Epidermal onion cells were directly transformed using the constructions made in pEMBL-GFP. Assays with the *X. laevis* embryos, HeLa and Drosophila S2 cells were monitored using the expression vector pCS2+ (Invitrogen), since the pCMV promoter is functional in vertebrate and insect cells [53]. All the transposase-GFP fusions were cloned at the *Stu*I and *Xba*I sites of pCS2+, using *Eco*RV-*Xba*I fragments purified from the seventeen fusions previously obtained in the pEMBL-GFP vectors. All the plasmids used for transfection were purified using the Qiagen Plasmid Midi Kit.

### Transformation of mammalian cells

HeLa cells were cultured in DMEM supplemented with 10% fetal bovine serum (FBS). Approximately 5×10^4^ cells were seeded onto each 24-well plate one day prior to transfection. Cells were transfected with transPEI, according to manufacturer's instructions (Eurogentec). Briefly, plasmid DNA (0.5 µg) and PEI (2 µl) were each diluted in 50 µl of 150 mM NaCl, and then mixed gently. After incubating for 15 min, the mixture was diluted with OPTIMEM medium to a final volume of 1 ml. Cells were then incubated with 0.1 ml of the complexes for 2 to 4 h. The transfection solution was then discarded and replaced by fresh DMEM supplemented with 10% FBS before being incubated for 24 hours at 37°C. Cells were observed under an epifluorescence microscope (Olympus BX51). The GFP fluorescence was imaged with a blue excitation filter set (460–490 nm excitation filter, 515 nm cut-off filter).

### Transformation of *Xenopus tropicalis* cells

*X. tropicalis* cells were propagated in L-15 medium diluted to 2/3 with sterile water, supplemented with 10% heat inactivated FBS and a cocktail of penicillin G (50 U/ml) and streptomycin (50 µg/ml). One day prior to transfection, 10^5^ cells were seeded on glass coverslips onto twelve-well plates in culture medium. Transfections were carried out using Lipofectamine™ LTX and PLUS™ reagent (Invitrogen) according to the manufacturer's instructions. One µl of PLUS™ reagent as well as 2 µl of Lipofectamine™ LTX transfection reagent were used to transfect 1 µg of each plasmid DNA. Two days after transfection, cells were fixed with 2% PFA in PBS for 20 min. The slides were then mounted in Mowiol-DAPI (4′, 6′-diamino-2-phenylindole, 500 ng/ml). Fluorescence of GFP (450–490 nm excitation filter, 510 nm cut-off filter) and DAPI (365 nm excitation filter, 395 nm cut-off filter) were imaged under a fluorescence microscope with a AxioCam MRm camera equipped with AxioVision software for image analysis (Zeiss, Germany).

### Transformation of insect cells

S2 cells were cultured in Schneider's Drosophila Medium supplemented with 10% FBS and P/S solution (50 U/ml penicillin and 50 U/ml streptomycin) in the absence of CO_2_ at 27°C. Five hours prior to transfection, about 2×10^6^ cells were seeded onto 24-well plates in medium without FBS or P/S. Cells were transfected with Cellfectin, according to the manufacturer's instructions (Invitrogen). Briefly, plasmid DNA (2 µg) and Cellfectin (5 µl) were diluted in 200 µl of medium, and gently mixed before being incubated for 15 min at room temperature. The mixture was then diluted with serum-free medium to a final volume of 400 µl. Cells were then incubated with 0.4 ml complexes for 3 h at 27°C. The transfection medium was discarded and replaced with fresh medium supplemented with 10% FBS. After incubating for 24 to 48 hours at 27°C, the cells were observed under an epifluorescence microscope (Olympus BX51). The GFP fluorescence was imaged with a blue excitation filter set (460–490 nm excitation filter, 515 nm cut-off filter).

### Biolistic transformation of onion epidermis cells

The surface of onions was disinfected with 70% ethanol. After dissection, samples of the internal epidermis were peeled and placed face up on solid vitamin-free MS medium (Duchefa, M0222) in 55-mm diameter Petri dishes. The particle bombardment transformation was carried out using a PDS-1000 Biorad system with 1800 psi rupture disks (Biorad) under reduced pressure (30 mm Hg). Ten µg of plasmid DNA was coated on tungsten M-25 particles (Biorad) in 25 µl of 2.5 M CaCl_2_ and 10 µl of 0.1 M spermidine. The particles were homogenized for 2 min by ultra-sonication and then sedimented under gravity for 15 min. Fifteen µl of the supernatant was removed and, after a short sonication step, 4 µl of the remaining particle mixture was placed on a macrocarrier disk (Biorad). During the bombardments, the samples were placed at a distance of 6 cm from the stopping screen. Onion cells were transformed with pEMBL-GFP carrying the various transposase-GFP fusions, as described [54], [55]. After transformation, the samples were incubated for 12 h in the dark at 25°C. Epidermal onion cells were observed under an epifluorescence microscope (Olympus BX51) equipped with a digital camera (Olympus DP50) and the corresponding software (Olympus Analysis). The GFP fluorescence was imaged with a blue excitation filter set (460–490 nm excitation filter, 515 nm cut-off filter).

### Controlled cellular localization GFP as detected by fluorescence microscopy

Three genes encoding GFP variants were kindly provided by Dr S. Kuijt (University Leiden, Netherlands). The first variant encoded GFP fused at its C-terminal end with a cardinal SV40 monopartite NLS (PKKKRKV). The second variant encoded GFP fused at its C-terminal end with a KDEL motif that addressed the protein to the endoplasmic reticulum. The third variant was specific to plant cells, and encoded a GFP fused at its N-terminal end with the RBCS-1A transit peptide that addresses proteins to the chloroplast. When calibrations were done using GFP alone in onion epidermis, HeLa, and *X. tropicalis* cells, and Drosophila S2, the GFP fluorescence signal was found in both the nucleus and the cytoplasm.

### Immunoblotting

Cells recovered from the cultures were washed three times with 1X PBS. Total protein extracts were separated by electrophoresis, adding 40 µg of each sample to a discontinuous sodium dodecyl sulfate 8% polyacrylamide mini-gel, and then electro-blotted onto nitrocellulose filters (Bio-Rad Laboratories). After blocking with 5% skim milk in phosphate-buffered saline for 1 h, the filters were incubated overnight with a mouse anti-V5 monoclonal antibody or a rabbit polyclonal anti-GFP (1∶2000 for both; invitrogen). The filters were then incubated with horseradish peroxidase-conjugated anti-rabbit IgG or anti-mouse IgG (Santa Cruz Biotechnology, Santa Cruz, CA) before being developed using enhanced chemiluminescence (Amersham Pharmacia Biotech, Sunnyvale, CA). Band intensities on the blot were measured using Image Gauge V3.45 software.

## Supporting Information

Supporting Information S1 GFP fluorescence patterns in *X. laevis* embryos microinjected with mRNA coding GFP and MOS1-GFP proteins.
(PDF)

Supporting Information S2 GFP fluorescence patterns of HIMAR1-GFP and MCMAR-GFP in mammal, amphibian, insect and plant cells. **S2a**: Analysis of the GFP-fluorescence of HIMAR1-FL GFP fusion. **S2b**: Analysis of the GFP-fluorescence of MCMAR1-FL GFP fusion.
(PDF)

Supporting Information S3 GFP fluorescence patterns in plant and insect cells transfected with MCMAR D3-GFP.
(PDF)

Supporting Information S4 Flow cytometric analysis of HeLa cells transfected with a plasmid expressing GFP.
(PDF)

Supporting Information S5 Sequence and expression properties of the optimized versions of the gene encoding MOS1.
(PDF)

Supporting Information S6 Oligonucleotides used in the studies.
(PDF)
