# Supplementary material for: Nuclear Importation of Mariner Transposases among Eukaryotes: Motif Requirements and Homo-Protein Interactions
Source: PLoS One. 2011 Aug 18;6(8):e23693. doi: 10.1371/journal.pone.0023693 (PMC3158080; doi:10.1371/journal.pone.0023693)
Supplement: Supporting Information S1 — GFP fluorescence patterns in X. laevis embryos microinjected with mRNA coding GFP and MOS1-GFP proteins. (PDF) [file pone.0023693.s001.pdf]

Nuclear Importation of *Mariner* Transposases among Eukaryotes: Motif Requirements, and Homo-Protein Interactions

Marie-Véronique Demattei, Sabah Hedhili, Ludivine Sinzelle, Christophe Bressac, Sophie Casteret, Nathalie Moiré, Jeanne Cambefort, Xavier Thomas, Nicolas Pollet, Pascal Gantet and Yves Bigot

Supporting Information 1:
GFP fluorescence patterns in *X. laevis* embryos microinjected with mRNA coding GFP and MOS1-GFP proteins

Figure legend - Features of the different fluorescence patterns observed in *X. laevis* embryos. Fluorescence pattern a was obtained when mRNA microinjection had been done with GFP alone, which diffuses into both compartments (DBC) due to the lack of any specific cellular addressing to the nucleus or cytoplasm. Fluorescence patterns c was obtained when mRNA microinjection had been done with a MOS1 FL-GFP fusion that is specifically addressed to the nucleus, due the presence of an NLS. Fluorescence patterns b and d are controls used to confirm the location of the nucleus by staining with Hoechst 33258 dye. The scale bar in d correspond to 100 µm.

Material and Methods

Transformation of X. laevis eggs by micro-injection

X. laevis eggs were obtained from females injected with human chorionic gonadotrophin. The eggs were artificially fertilized and dejellied as described[1].

Xenopus laevis eggs were obtained from adult females (a strain maintained at UMR 8080, Orsay - France) by injected with human chorionic gonadotrophin. *X. laevis* embryos were obtained by *in-vitro* fertilization using standard methods. Embryos were staged according to the Nieuwkoop and Faber table of development.

Capped RNAs were prepared from NotI-linearized pCS2 vectors using mMessage mMachine kit (Ambion). The RNAs were injected in a volume of 9.2 nl and at a concentration of 100 pg/nl into one-cell stage embryos using a nanojet injector. Embryos were staged as described[2].

Microinjected embryos were raised up to the blastula stage, and then the animal caps were dissected out for observation and scoring using an Olympus SZX12 microscope with fluorescent illumination. Brightfield and GFP images were obtained using a color video camera (Spot insight color, Diagnostic instrument) with a 460- to 490-nm excitation filter and a 510-nm cut-off filter.

References

1. Sive HL, Grainger R, Harland R (2000) Early development of *Xenopus laevis* : a laboratory manual. Cold Spring Harbor Laboratory Press, New York.

2. Nieuwkoop PD & Faber P (1967) Normal table of *Xenopus laevis* (Daudin). 2nd ed., Elsevier North Holland Publishing Company, Amsterdam.

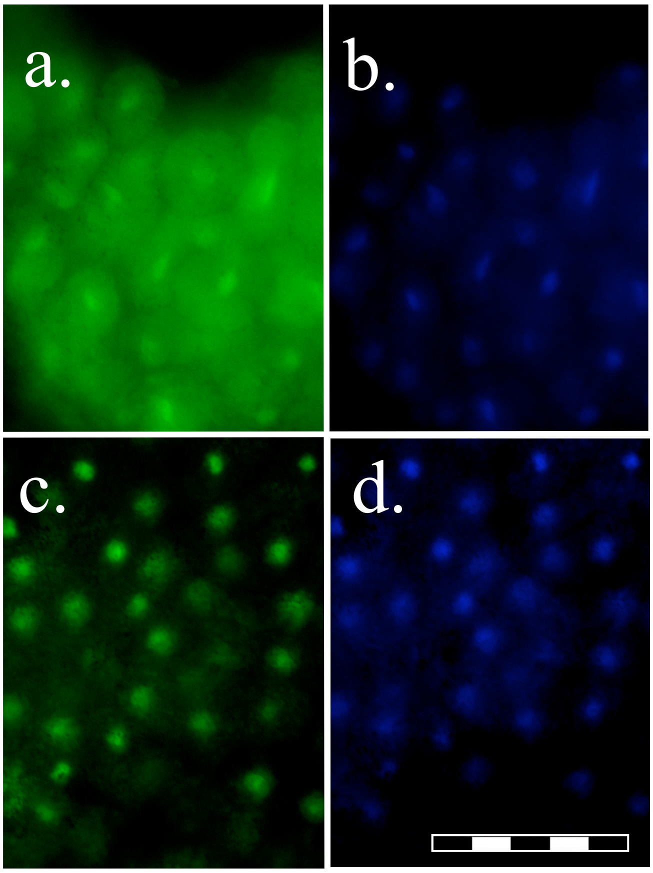
a.
b.
c.
d.
