# Supplementary material for: Nuclear Importation of Mariner Transposases among Eukaryotes: Motif Requirements and Homo-Protein Interactions
Source: PLoS One. 2011 Aug 18;6(8):e23693. doi: 10.1371/journal.pone.0023693 (PMC3158080; doi:10.1371/journal.pone.0023693)

Nuclear Importation of *Mariner* Transposases among Eukaryotes: Motif Requirements, and Homo-Protein Interactions

Marie-Véronique Demattei, Sabah Hedhili, Ludivine Sinzelle, Christophe Bressac, Sophie Casteret, Nathalie Moiré, Jeanne Cambefort, Xavier Thomas, Nicolas Pollet, Pascal Gantet and Yves Bigot

Supporting Information 2 :
GFP fluorescence patterns of HIMAR1-GFP and MCMAR-GFP in mammal, amphibian, insect and plant cells

Figure legends -

a. Analysis of the GFP-fluorescence of HIMAR1-FL GFP fusion in human HeLa cells, *X. tropicalis* cells, insect cells, and onion epidermal cells. The left panels show GFP fluorescence, the middle panels show the nuclear genomic DNA staining by DAPI, the right panels correspond to merge pictures.

b. Analysis of the GFP-fluorescence of MCMAR1-FL GFP fusion in human HeLa cells, *X. tropicalis* cells, insect cells, and onion epidermal cells. The left panels show GFP fluorescence, the middle panels show the nuclear genomic DNA staining by DAPI, the right panels correspond to merge pictures.

a. HIMAR1-GFP fusion

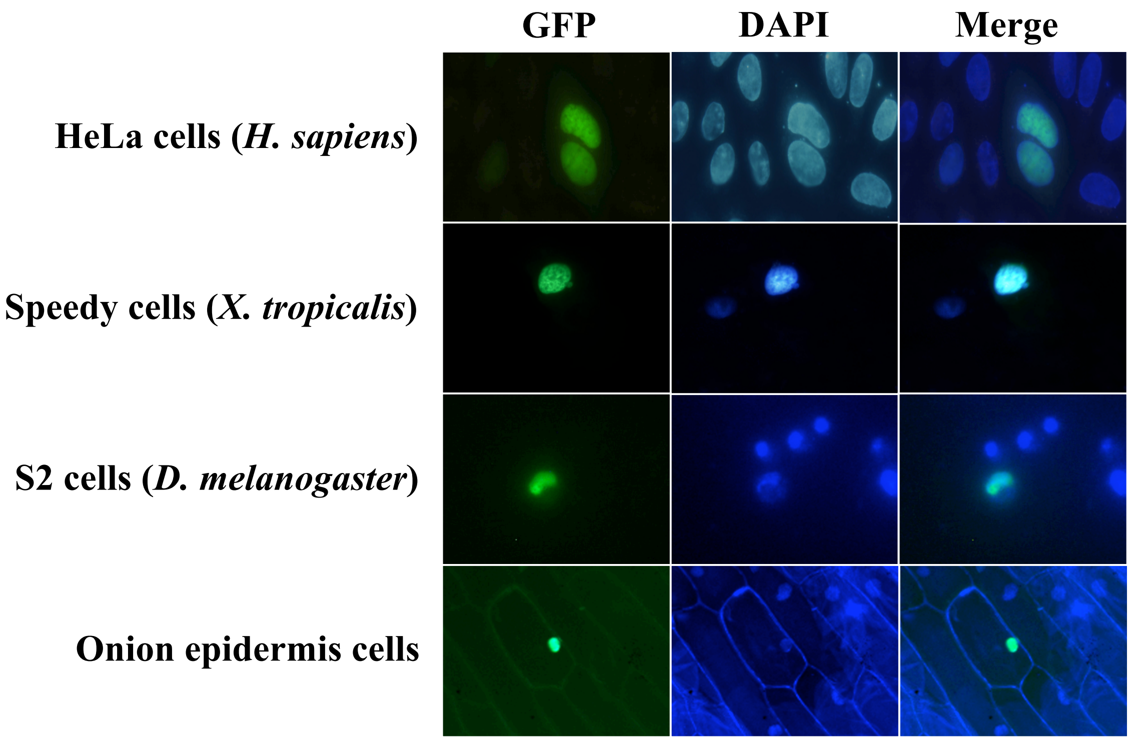

b. MCMAR1-GFP fusion

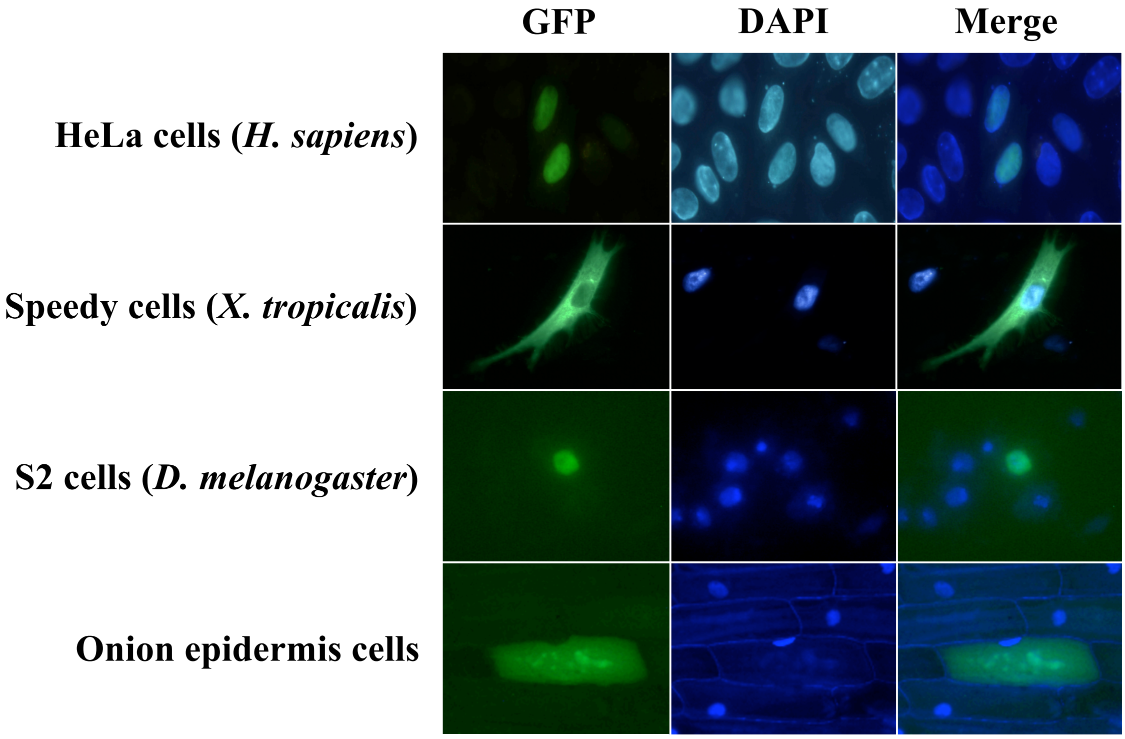

Supplement: Supporting Information S2 — GFP fluorescence patterns of HIMAR1-GFP and MCMAR-GFP in mammal, amphibian, insect and plant cells. S2a: Analysis of the GFP-fluorescence of HIMAR1-FL GFP fusion. S2b: Analysis of the GFP-fluorescence of MCMAR1-FL GFP fusion. (PDF) [file pone.0023693.s002.pdf]
