# Supplementary material for: Nuclear Importation of Mariner Transposases among Eukaryotes: Motif Requirements and Homo-Protein Interactions
Source: PLoS One. 2011 Aug 18;6(8):e23693. doi: 10.1371/journal.pone.0023693 (PMC3158080; doi:10.1371/journal.pone.0023693)
Supplement: Supporting Information S3 — GFP fluorescence patterns in plant and insect cells transfected with MCMAR D3-GFP. (PDF) [file pone.0023693.s003.pdf]

Nuclear Importation of *Mariner* Transposases among Eukaryotes: Motif Requirements, and Homo-Protein Interactions

Marie-Véronique Demattei, Sabah Hedhili, Ludivine Sinzelle, Christophe Bressac, Sophie Casteret, Nathalie Moiré, Jeanne Cambefort, Xavier Thomas, Nicolas Pollet, Pascal Gantet and Yves Bigot

Supporting Information 3 :
GFP fluorescence patterns in plant and insect cells transfected with MCMAR Δ3-GFP

Figure legend - Features of the different fluorescence patterns observed in onion epidermal cells (a to c) and in Drosophila S2 cells (d to f). Fluorescence patterns a and d were obtained when cells had been transfected with GFP alone, which diffuses into both compartments (DBC) due to the lack of any specific cellular addressing to the nucleus or cytoplasm. Fluorescence patterns b and e were obtained when cells had been transfected with a GFP fusion that is specifically addressed to the nucleus, due the presence of an SV40 NLS fused at the C-terminal end of the GFP. Fluorescence patterns c and f were obtained when cells expressed an MCMAR1 Δ3-GFP.

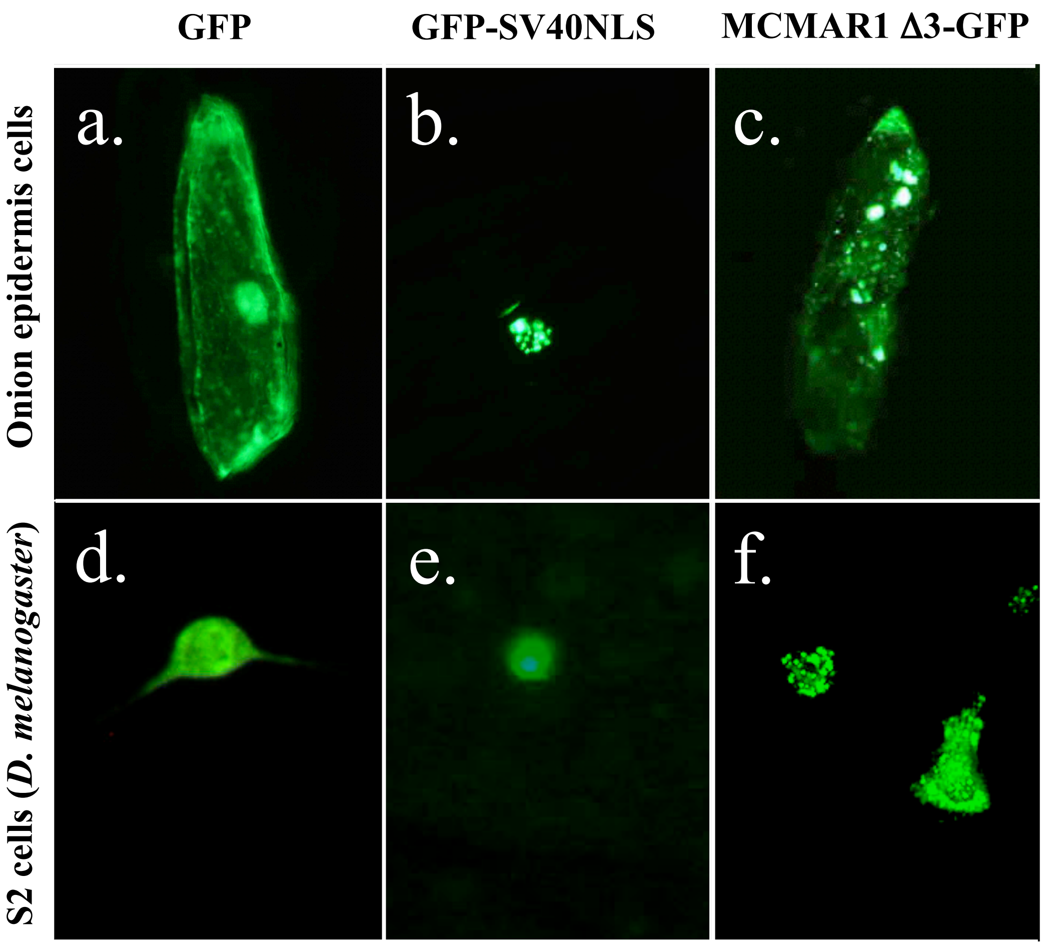
GFP
GFP-SV40NLS
MCMAR1 Δ3-GFP
Onion epidermis cells
S2 cells (*D. melanogaster*)
a.
b.
c.
d.
e.
f.
