# Supplementary material for: Nuclear Importation of Mariner Transposases among Eukaryotes: Motif Requirements and Homo-Protein Interactions
Source: PLoS One. 2011 Aug 18;6(8):e23693. doi: 10.1371/journal.pone.0023693 (PMC3158080; doi:10.1371/journal.pone.0023693)
Supplement: Supporting Information S4 — Flow cytometric analysis of HeLa cells transfected with a plasmid expressing GFP. (PDF) [file pone.0023693.s004.pdf]

Nuclear Importation of *Mariner* Transposases among Eukaryotes: Motif Requirements, and Homo-Protein Interactions

Marie-Véronique Demattei, Sabah Hedhili, Ludivine Sinzelle, Christophe Bressac, Sophie Casteret, Nathalie Moiré, Jeanne Cambefort, Xavier Thomas, Nicolas Pollet, Pascal Gantet and Yves Bigot

Supporting Information 4 : Flow cytometric analysis of HeLa cells transfected with a plasmid expressing GFP.

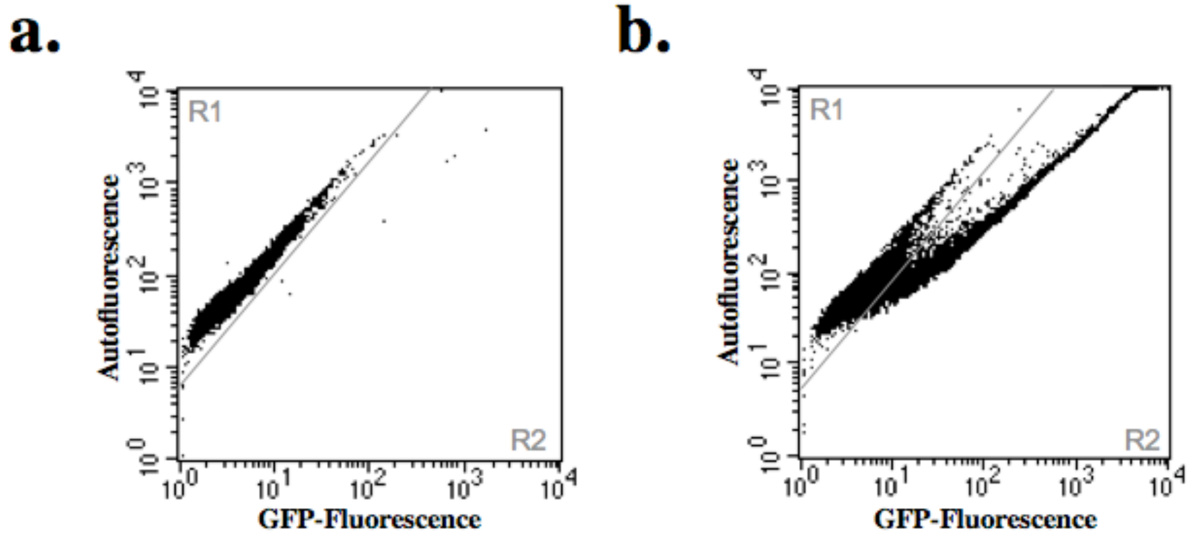

Legend. Flow cytometry analyses of cells transfected with the calibration controls pCS2 (a) and pCS2-GFP (b), taking into account the GFP-fluorescence (horizontal axis) and auto-fluorescence (vertical axis) of the cells. Cells expressing and not expressing GFP were contained in gates R1 and R2, respectively. We observed that the GFP-fluorescent signal between cells varied by up to 3.5 orders of magnitude.

Material and methods.

Transformation of mammalian cells

HeLa cells were cultured in DMEM supplemented with 10% fetal bovine serum. About 8 x 10^4 cells were seeded onto a 24-well plate one day prior to transfection. The cells were transfected with TransPEI, according to the Manufacturer's instructions (Eurogentec). Briefly, plasmid DNA (0.5 µg) and TransPEI (1 µl) were separately diluted with 50 µl of 150 mM NaCl solution, and then gently mixed. After incubating for 30 min, the mixture was diluted in OPTIMEM medium to a final volume of 1 ml. The cells were then incubated with 0.1 ml complexes for 2 to 4 h. The transfection solution was then removed and replaced with fresh supplemented DMEM, and the cells were then incubated for 24 hours at 37°C.

Flow cytometry

Cells recovered from the cultures 24-hours post transfection were washed three times with 1X PBS. The cell pellet was finally resuspended in 400 µl 1X PBS supplemented with 2% paraformaldehyde (w/v), and stored at 4°C. The analyses were performed using a flow cytometer FACSORT and the Cell Quest program (Beckton Dickinson). A total of 20,000 cells were acquired for each sample. Dead cells and debris were excluded from the analysis based on forward angle and side scatter light gating. Analysis gates were determined from the Green fluorescence intensity using transfection controls done with plasmids expressing or not expressing GFP.
