# Supplementary material for: Nuclear Importation of Mariner Transposases among Eukaryotes: Motif Requirements and Homo-Protein Interactions
Source: PLoS One. 2011 Aug 18;6(8):e23693. doi: 10.1371/journal.pone.0023693 (PMC3158080; doi:10.1371/journal.pone.0023693)
Supplement: Supporting Information S5 — Sequence and expression properties of the optimized versions of the gene encoding MOS1. (PDF) [file pone.0023693.s005.pdf]

Nuclear Importation of *Mariner* Transposases among Eukaryotes: Motif Requirements, and Homo-Protein Interactions

Marie-Véronique Demattei, Sabah Hedhili, Ludivine Sinzelle, Christophe Bressac, Sophie Casteret, Nathalie Moiré, Jeanne Cambefort, Xavier Thomas, Nicolas Pollet, Pascal Gantet and Yves Bigot

Supporting Information 5 : Sequence and expression properties of the optimized versions of the gene encoding MOS1

In silico *design of optimized MOS1 ORF*

Previous studies have demonstrated that the expression capacities of the eukaryotic genes can be restricted by several non-mutually exclusive factors[1,2]. The first such factor is the ability of the mRNA to be accurately translated by the cellular machinery. Indeed, we observed that most of the MLE transposase ORFs available in databanks and in the literature had no Kozak box overlapping their start codon, thus potentially reducing the ability of the ribosomes to initiate the efficient translation of the transcript into protein. We also observed that most MLE transposase ORFs contained high levels of rare codons. For example, we found 39 rare codons in the ORF encoding MOS1 regardless of whether this protein was expressed in human or in Drosophila cells, the host from which it was cloned (Fig. 1, lane 1). These observations therefore supported the hypothesis that the translation of MLE transposase mRNA could be limited in many eukaryotic cells by codon usage, as previously demonstrated for other proteins[3]. These facts were used to define a first version of the MOS1 ORF, MOS1V1, which included a Kozak box and an optimal codon usage for protein expression in human cells (Fig. 1, lane 2).

The second kind of factor that might reduce MOS1 expression involved an intrastrand property of its ORF. We have previously shown[4,5] that two elements belonging to the *mauritiana* sub-family, *Mos1* and *Botmar1*, both have a nucleic acid sequence that is able to form similar intrastrand DNA structures, despite their sequence divergence (25%). The increase in the sequence data available in databanks has allowed us to confirm with four other MLEs belonging to the *mauritiana* subfamily (*Momar1* (Acc No.: U15665), *Mdmar1* (Acc N°: U24436), *Ramar1* (Acc N°: DQ784570.1) and MEBOTRA1.4-9 (Acc No.: AJ781770.1)) that this property was conserved, whatever the nucleic acid sequence divergence (25-45%; data not shown). If such intrastrand

structures can be stably annealed within the genomic DNA, then they could restrict transcription into mRNA. If they occur in mRNA, they could inhibit their translation into protein by two non-mutually exclusive pathways. First, they could directly block ribosome function. Second, they could elicit the RNA-silencing, since the large intrastrand stem-loop structures might be recognized by *Dicer*-like proteins as potential substrates for making small interfering RNA molecules 20-30 nucleotides in length. Such an RNA self-silencing mechanism would be then able to erase the MLE transcripts.

To circumvent these potential problems of intrastrand annealing, we investigated the significance of the structures obtained in the wild-type *Mos1* ORF (MOS1) and the MOS1V1 using a previously devised statistical method[5]. The resulting graphs showed that all intrastrand folds obtained with MOS1 and most of those obtained with MOS1V1 were statistically consistent (Figure 2 a,b). Since a high proportion of the stem-loop structures was conserved between the folds obtained for both nucleic acid sequences, we removed them manually from the stem regions of the MOS1V1 using the degeneracy of the codon usage to design a second version of the MOS1 ORF, MOS1V2 (Fig. 1, lane 3). In this second version, we obviously avoided using rare codons to eliminate secondary structure. The statistical significance of the intrastrand folds obtained with MOS1V2 was verified. Graphs of the results (Figure 2c) showed that they were not significant, since they were all located in the middle of the space defined by the density ellipses. The nucleic acid features of the MOS1, MOS1V1 and MOSV2 ORFs are summarized in Table 1.

Table 1 – DNA sequence properties of the three versions of the MOS1 ORF

ORF	ORF length	GC content	Number of CpG dinucleotides	Percentage of sequence similarity		
				MOS1	MOS1V1	MOS V2
**MOS1**	1038	46.52	64	100	74.1	74.8
**MOS1V1**	1038	66.18	140	74.1	100	93.5
**MOS1V2**	1038	62.03	111	74.8	93.5	100

The third kind of factor that could affect MLE transposase expression concerned the stability of the mRNA transcripts. It was recently demonstrated that adding specific 5' and 3' untranslated terminal regions (UTRs) has the effect of markedly increasing the expression of the *Sleeping Beauty* and *Himar1* transposases[6,7]. In an attempt to find out whether the presence of such UTRs interfered with the expression of MOS1 in HeLa cells, we constructed three expression plasmids in which MOS1 [+], MOSV1 and MOS1V2 were flanked by the β-globin 5' and 3' UTRs.

Expression of the different MOS1 ORFs in HeLa cells

In an attempt to track the expression in HeLa cells, the three MOS1 versions were fused at their C-terminal end with GFP, and then cloned in the expression vector pCS2+, as described in the core manuscript. The resulting three expression plasmids were designated MOS1-GFP, MOS1V1-GFP, and MOS1V2-GFP respectively. Three complementary constructs were made to investigate the impact of the UTR, and designated U5-MOS1-GFP-U3, U5-MOS1V1-GFP-U3 and U5-MOS1V2-GFP-U3 respectively. Finally, two expression plasmids, designated pCS2 and pCS2-GFP, were used as negative and positive expression controls respectively. The expression pattern of the various GFP fusions was investigated by western blot analyses of crude protein extracts of plasmid-transfected cell hybridized with anti-GFP antibodies. Our results indicated that the amounts of GFP fusion produced in cells transfected with the MOS1V2-GFP and U5-MOS1V2-GFP-U3 plasmids were 12 to 15-fold higher than those produced with the MOS1-GFP plasmid (Fig. 3).

Materials and Methods

Sequence analyses and statistical treatments

Analyses of the intrastrand DNA structure in the cDNA sequence used the Mfold program[8] from the web site http://bioweb.pasteur.fr/seqanal/tmp/mfold/. The statistical analyses of the significance of the folds obtained for each MLE sequence were carried out by creating files of 1000 shuffled sequences with the shuffleseq program at the website http://emboss.bioinformatics.nl/. To calculate the folds of each shuffled sequence, the stand-alone version 3.2 of Mfold (Red Hat binaries) was downloaded. In order to match the physiological conditions of HeLa cells, the folds were computed at 37°C using default parameters. We observed that the main sequence segments found to be involved in intrastrand annealings of the MOS1 ORF at 28°C[5] were the same whether the calculations were done at 30° or at 37°C. Computing was done on a Mac OS X version 10.4.8 equipped with 2 Go RAM and 2 Dual-Core Intel Xeon 2.66 GHz. A bash script was used to analyze all sets of 1000 sequences. The number of folds and their ΔG for each sequence were recovered, and their graphic statistical analysis was computed by calculating their nonparametric bivariate density using the JMP version 3.6 package available at http://www.jmp.com/.

The codon usage of the various putative hosts of the MLE was investigated using data available at http://www.kazusa.or.jp/codon/. It was optimized using facilities of the Jcat software at http://www.prodoric.de/JCat/.

DNA sources

The ORF encoding the original natural *Mos1* transposase (MOS1; Acc N°**X78906**) was recovered as described elsewhere[9]. The two versions of the MOS1 ORF (MOS1V1 and MOS1V2)

with the optimized nucleic acid sequences, MOS1V1 and MOS1V2, were synthesized by ATG:Biosynthetics (Germany).

Plasmid constructs

The green fluorescent protein (GFP) cassette fused at the C-terminal ends of the three transposase fragments originated from the pCAMBIA-1302 (Acc No.: AF134298) as described in the Material and Methods section of the main manuscript. It was cloned as a *Eco*RI-*Xba*I fragment at the *Stu*I and *Xba*I sites of the multicloning site of the expression vector pCS2+ (Invitrogen; pCS2-GFP). Cloning for the fusion of the various MOS1 ORFs with GFP was done at the *Spe*I site (pCS2-MOS1-GFP, pCS2-MOS1V1-GFP, pCS2-MOS1V2-GFP). The ORFs encoding the GFP fusions with the three MOS1 versions were also cloned in a pCS2+ vector, in which they were flanked by the 5' and 3' UTRs of the *Xenopus laevis* β-globin mRNA.

Transformation of mammalian cells

HeLa cells were cultured in DMEM supplemented with 10% fetal bovine serum. About 8 x 10^4 cells were seeded onto a 24-well plates, one day prior to transfection. The cells were transfected with TransPEI, according to the Manufacturer's instructions (Eurogentec). Briefly, plasmid DNA (0.5 μg) and TransPEI (1 μl) were separately diluted into 50 μl of 150 mM NaCl solution and then gently mixed together. After incubating for 30 min, the mixture was diluted in OPTIMEM medium to a final volume of 1 ml. The cells were then incubated with 0.1 ml complexes for 2 to 4 h. The transfection solution was then removed and replaced with fresh supplemented DMEM and the cells were incubated for 24 hours at 37°C. Cells were observed with an epifluorescence microscope (Olympus BX51). The GFP fluorescence was imaged with a blue excitation filter set (460-490 nm excitation filter, 515 nm cut-off filter).

Immunoblotting

Cells recovered from the cultures were washed three times with 1X PBS. Total protein extracts were separated by electrophoresis, adding 40 μg of each sample to a discontinuous sodium dodecyl sulfate 8% polyacrylamide mini-gel, and then electro-blotted onto nitrocellulose filters (Bio-Rad Laboratories). After blocking with 5% skim milk in phosphate-buffered saline for 1 h, the filters were incubated overnight with rabbit polyclonal anti-GFP (1: 2000; invitrogen). The filters were then incubated with horseradish peroxidase-conjugated anti-rabbit IgG (Santa Cruz Biotechnology, Santa Cruz, CA) before being developed using enhanced chemiluminescence (Amersham Pharmacia Biotech, Sunnyvale, CA). Band intensities on the blot were measured using Image Gauge V3.45 software.

```
                    M   S   S   F   V   P   N   K   E   Q   T   R   T   V   L   I      16
[+] tcagtgcagtcaac ATG TCG AGT TTC GTG CCG AAT AAA GAG CAA ACG CGG ACA GTA TTA ATT   222
V1  tcagtgcagtcaac ATG AGC AGC TTC GTG CCC AAC AAG GAG CAG ACC CGC ACC GTG CTG ATC
V2  tcagtgcagtcaac ATG TCC TCC TTC GTG CCC AAC AAG GAG CAG ACC CGC ACC GTG CTG ATC

     F   C   F   H   L   K   K   T   A   A   E   S   H   R   M   L   V   E   A   F    36
[+] TTC TGT TTT CAT TTG AAG AAA ACA GCT GCG GAA TCG CAC CGA ATG CTT GTT GAA GCC TTT   282
V1  TTC TGC TTC CAC CTG AAG AAG ACC GCC GCC GAG AGC CAC CGC ATG CTG GTG GAG GCC TTC
V2  TTT TGT TTT CAC CTG AAG AAG ACC GCC GCC GAG TCC CAC CGC ATG CTG GTG GAG GCC TTC

     G   E   Q   V   P   T   V   K   T   C   E   R   W   F   Q   R   F   K   S   G    56
[+] GGC GAA CAA GTA CCA ACT GTG AAA ACG TGT GAA CGG TGG TTT CAA CGC TTC AAA AGT GGT   342
V1  GGC GAG CAG GTG CCC ACC GTG AAG ACC TGC GAG CGC TGG TTC CAG CGC TTC AAG AGC GGC
V2  GGA GAG CAG GTG CCT ACC GTG AAG ACA TGT GAA CGG TGG TTC CAG CGC TTC AAG AGC GGA

     D   F   D   V   D   D   K   E   H   G   K   P   P   K   R   Y   E   D   A   E    76
[+] GAT TTT GAC GTC GAC GAC AAA GAG CAC GGA AAA CCG CCA AAA AGG TAC GAA GAC GCC GAA   402
V1  GAC TTC GAC GTG GAC GAC AAG GAG CAC GGC AAG CCC CCC AAG CGC TAC GAG GAC GCC GAG
V2  GAC TTT GAC GTG GAC GAC AAG GAG CAT GGC AAG CCC CCC AAG CGT TAC GAG GAC GCC GAG

     L   Q   A   L   L   D   E   D   D   A   Q   T   Q   K   Q   L   A   E   Q   L    96
[+] CTG CAA GCA TTA TTG GAT GAA GAC GAT GCT CAA ACG CAA AAA CAA CTC GCA GAG CAG TTG   462
V1  CTG CAG GCC CTG CTG GAC GAG GAC GAC GCC CAG ACC CAG AAG CAG CTG GCC GAG CAG CTG
V2  CTG CAG GCT CTG CTG GAC GAG GAC GAC GCT CAG ACC CAG AAG CAG CTG GCA GAA CAG CTG

     E   V   S   Q   Q   A   V   S   N   R   L   R   E   M   G   K   I   Q   K   V   116
[+] GAA GTA AGT CAA CAA GCA GTT TCC AAT CGC TTG CGA GAG ATG GGA AAG ATT CAG AAG GTC   522
V1  GAG GTG AGC CAG CAG GCC GTG AGC AAC CGC CTG CGC GAG ATG GGC AAG ATC CAG AAG GTG
V2  GAG GTG AGC CAG CAG GCC GTG AGC AAC CGC CTG CGC GAG ATG GGC AAG ATC CAG AAG GTG

     G   R   W   V   P   H   E   L   N   E   R   Q   M   E   R   R   K   N   T   C   136
[+] GGT AGA TGG GTG CCA CAT GAG TTG AAC GAG AGG CAG ATG GAG AGG CGC AAA AAC ACA TGC   582
V1  GGC CGC TGG GTG CCC CAC GAG CTG AAC GAG CGC CAG ATG GAG CGC CGC AAG AAC ACC TGC
V2  GGC CGC TGG GTG CCC CAC GAG CTG AAC GAG CGC CAG ATG GAG CGC CGC AAG AAC ACC TGC

     E   I   L   L   S   R   Y   K   R   K   S   F   L   H   R   I   V   T   G   D   156
[+] GAA ATT TTG CTT TCA CGA TAC AAA AGG AAG TCG TTT TTG CAT CGT ATC GTT ACT GGA GAT   642
V1  GAG ATC CTG CTG AGC CGC TAC AAG CGC AAG AGC TTC CTG CAC CGC ATC GTG ACC GGC GAC
V2  GAG ATC CTG CTG AGC CGC TAC AAG CGC AAG AGC TTC CTG CAC CGC ATC GTG ACC GGC GAC

     E   K   W   I   F   F   V   N   P   K   R   K   K   S   Y   V   D   P   G   Q   176
[+] GAA AAA TGG ATC TTT TTT GTT AAT CCT AAA CGT AAA AAG TCA TAC GTT GAT CCT GGA CAA   702
V1  GAG AAG TGG ATC TTC TTC GTG AAC CCC AAG CGC AAG AAG AGC TAC GTG GAC CCC GGC CAG
V2  GAG AAG TGG ATT TTT TTT GTG AAC CCC AAG CGC AAG AAG TCC TAC GTG GAC CCA GGA CAG

     P   A   T   S   T   A   R   P   N   R   F   G   K   K   T   M   L   C   V   W   196
[+] CCG GCC ACA TCG ACT GCT CGA CCG AAT CGC TTT GGC AAG AAG ACG ATG CTC TGT GTT TGG   762
V1  CCC GCC ACC AGC ACC GCC CGC CCC AAC CGC TTC GGC AAG AAG ACC ATG CTG TGC GTG TGG
V2  CCT GCT ACA TCC ACA GCT CGC CCT AAC CGC TTT GGA AAG AAG ACC ATG CTG TGC GTG TGG

     W   D   Q   S   G   V   I   Y   Y   E   L   L   K   P   G   E   T   V   N   T   216
[+] TGG GAT CAG AGC GGT GTC ATT TAC TAT GAG CTC TTG AAA CCC GGC GAA ACG GTG AAT ACG   822
V1  TGG GAC CAG AGC GGC GTG ATC TAC TAC GAG CTG CTG AAG CCC GGC GAG ACC GTG AAC ACC
V2  TGG GAC CAG TCC GGA GTG ATC TAC TAC GAG TTG TTG AAG CCC GGA GAG ACC GTG AAC ACC

     A   R   Y   Q   Q   Q   L   I   N   L   N   R   A   L   Q   R   K   R   P   E   236
[+] GCA CGC TAC CAA CAA CAA TTG ATC AAT TTG AAC CGT GCG CTT CAG AGA AAA CGA CCG GAA   882
V1  GCC CGC TAC CAG CAG CAG CTG ATC AAC CTG AAC CGC GCC CTG CAG CGC AAG CGC CCC GAG
V2  GCC CGC TAC CAG CAG CAG CTG ATT AAC CTG AAC CGC GCC CTG CAG CGG AAG CGC CCC GAG

     Y   Q   K   R   Q   H   R   V   I   F   L   H   D   N   A   P   S   H   T   A   256
[+] TAT CAA AAA AGA CAA CAC AGG GTC ATT TTT CTC CAT GAC AAC GCT CCA TCA CAT ACG GCA   942
V1  TAC CAG AAG CGC CAG CAC CGC GTG ATC TTC CTG CAC GAC AAC GCC CCC AGC CAC ACC GCC
V2  TAC CAG AAG CGC CAG CAT CGC GTG ATC TTC CTG CAC GAC AAT GCC CCT AGC CAC ACC GCC

     R   A   V   R   D   T   L   E   T   L   N   W   E   V   L   P   H   A   A   Y   276
[+] AGA GCG GTT CGC GAC ACG TTG GAA ACA CTC AAT TGG GAA GTG CTT CCG CAT GCG GCT TAC 1002
V1  CGC GCC GTG CGC GAC ACC CTG GAG ACC CTG AAC TGG GAG GTG CTG CCC CAC GCC GCC TAC
V2  CGC GCC GTG CGG GAC ACC CTG GAG ACC CTG AAC TGG GAG GTG CTG CCC CAC GCC GCC TAC

     S   P   D   L   A   P   S   D   Y   H   L   F   A   S   M   G   H   A   L   A   296
[+] TCA CCA GAC CTG GCC CCA TCC GAT TAC CAC CTA TTC GCT TCG ATG GGA CAC GCA CTC GCT 1062
V1  AGC CCC GAC CTG GCC CCC AGC GAC TAC CAC CTG TTC GCC AGC ATG GGC CAC GCC CTG GCC
V2  AGC CCC GAC CTG GCC CCC AGC GAC TAC CAC CTG TTC GCC TCC ATG GGA CAC GCC CTG GCC

     E   Q   R   F   D   S   Y   E   S   V   K   K   W   L   D   E   W   F   A   A   316
[+] GAG CAG CGC TTC GAT TCT TAC GAA AGT GTG AAA AAA TGG CTC GAT GAA TGG TTC GCC GCA 1122
V1  GAG CAG CGC TTC GAC AGC TAC GAG AGC GTG AAG AAG TGG CTG GAC GAG TGG TTC GCC GCC
V2  GAG CAG CGC TTT GAC TCC TAC GAG TCC GTG AAG AAG TGG CTG GAC GAG TGG TTT GCC GCT

     K   D   D   E   F   Y   W   R   G   I   H   K   L   P   E   R   W   E   K   C   336
[+] AAA GAC GAT GAG TTC TAC TGG CGT GGA ATC CAC AAA TTG CCC GAG AGA TGG GAA AAA TGT 1182
V1  AAG GAC GAC GAG TTC TAC TGG CGC GGC ATC CAC AAG CTG CCC GAG CGC TGG GAG AAG TGC
V2  AAG GAC GAC GAG TTC TAC TGG CGC GGA ATC CAC AAG CTG CCC GAG CGG TGG GAG AAA TGC

     V   A   S   D   G   K   Y   F   E                                             345
[+] GTA GCT AGC GAC GGC AAA TAC TTT GAA  taaatgatttttttctttttccacaaaatttaacgtgtttttt 1251
V1  GTG GCC AGC GAC GGC AAG TAC TTC GAG  taaatgatttttttctttttccacaaaatttaacgtgtttttt
V2  GTG GCC TCC GAC GGC AAG TAC TTC GAG  taaatgatttttttctttttccacaaaatttaacgtgtttttt
```

Figure 1. Nucleic acid sequence alignment of the natural MOS1 ORF (lane [+]), the first version of MOS1 ORF, MOS1V1, in which is included a Kozak box and optimal codons for an expression in human cells, (lane V1), and the second version of MOS1 ORF, MOS1V2, that corresponds to a version of MOS1V1 in which putative secondary structures have been removed (lane V2). The amino acid sequence of MOS1 is shown above sequence alignment. Codons that are rare in the human codon usage are highlighted in gray in the MOS1 ORF. The Kozak box at the 5' end of V1, and the stop codons at the 3' ends are boxed. Sequence regions in lower-case letters at both sequence ends correspond to *Mos1* UTR regions. Putative ARE and ARE-like motifs are underlined. Numbers in italics in the right margin indicate the amino acid positions. Those in roman numerals correspond to the nucleic acid positions in the original *Mos1* transposon sequence (Acc N°: **X78906**).

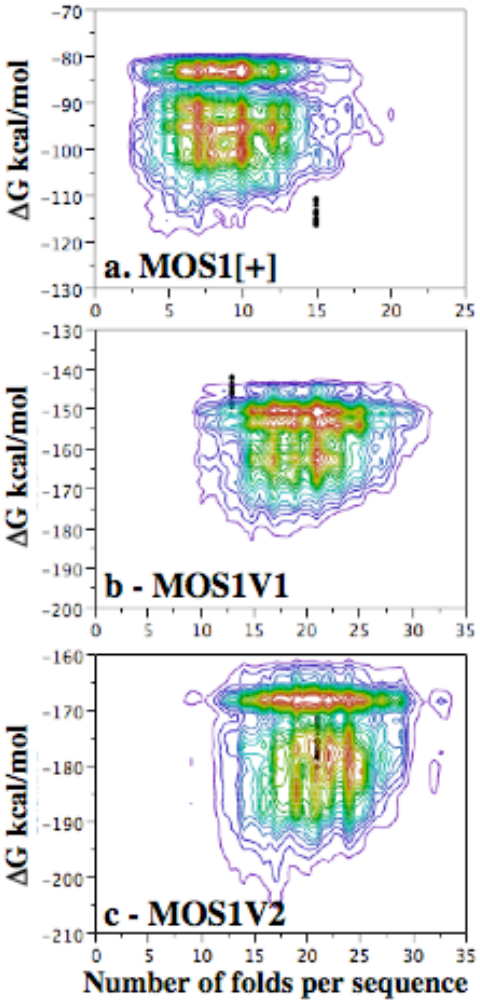

Figure 2. Graphic analysis of the statistical significance of the secondary structures calculated using the nucleic acid sequences shown in Figure 1 for the natural MOS1 ORF (a), and for MOS1V1 (b), and MOS1V2 (c). For each sequence, the number of folds (horizontal axis) was used to posit the entropy value (vertical axis) of each of its folds. Files containing 1000 scrambled DNA sequences similar to the nucleotide composition of each investigated sequence were first used to calculate the folds, using Mfold, and then the results were used to calculate the nonparametric bivariate density ellipse. 21949, 37637 and 36517 spots were obtained to calculate the ellipses in a, b and c respectively. These ellipses depicted the lack of significance of the secondary structures obtained with non-structured sequences in concentric distribution spaces ranging from 100% (red) to 0% (blue), in steps of 5%. Each of these spaces was delineated by thin or thick colored lines. Since these spaces were calculated from non-structured sequences, they also described probability spaces that can be used to locate structured or non-structured folds. The space describing an absence of significance is delineated in each graphic with a threshold of 90% by the third ellipse in blue from the outside. The folds obtained with each MLE sequence are indicated by small black squares.

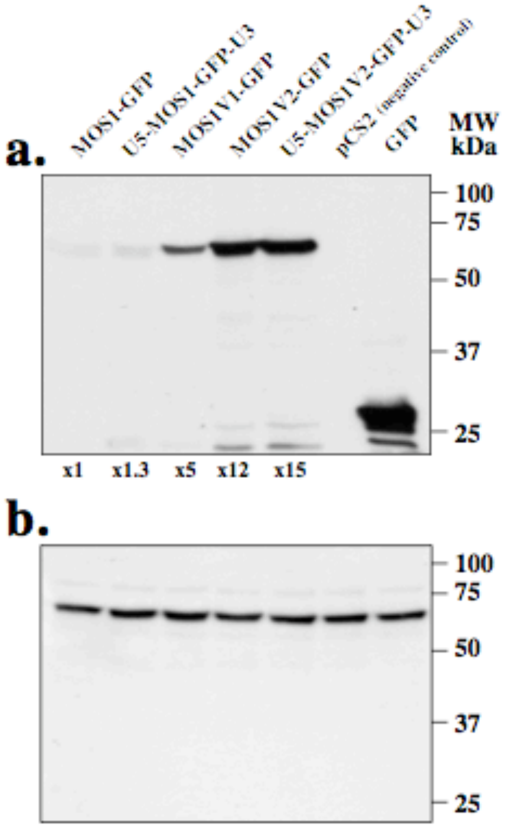

Figure 3. Western blot analysis of GFP fusion expressions in HeLa cells. GFP fusions were revealed by first hybridizing a rabbit polyclonal anti-GFP (1 : 2000) in (a). Then the filters were incubated with horseradish peroxidase-conjugated anti-rabbit IgG, followed by development using enhanced chemiluminescence. Values below the pictures indicate the increase in expression of each GFP fusion compared to MOS1-GFP. (b) shows an expression control with the housekeeping protein encoded by the MEN1 gene[10] that was revealed by hybridizing a rabbit polyclonal anti-MEN1, and then incubating with horseradish peroxidase-conjugated anti-rabbit IgG, followed by development using enhanced chemiluminescence.

References

1. Cherepanov, P., Pluymers, W., Claeys, A., Proost, P., De Clercq , E. & Debyser Z. (2000) High-level expression of active HIV-1 integrase from a synthetic gene in human cells. *FASEB J.* **14**, 1389-99.
2. Guhaniyogi, J. & Brewer, G. (2001) Regulation of mRNA stability in mammalian cells. *Gene*, **265**, 11-23.
3. Yant, S.R., Huang, Y., Akache, B. & Kay, M.A. (2007) Site-directed transposon integration in human cells. *Nucl. Acids Res.* 35:e50.
4. Rouleux-Bonnin, F., Petit, A., Demattei, M.V., and Bigot, Y. (2005) Evolution of full-length and deleted forms of the *mariner*-like element, *Botmar1*, in the Genome of the bumble bee, *Bombus terrestris* (Hymenoptera: Apidae). *J. Mol. Evol.* **60**, 736-747.
5. Petit, A., Rouleux-Bonnin, F., Lambelé, M., Pollet, N. & Bigot, Y. (2007) Properties of the various *Botmar1* transcripts in imagoes of the bumble bee, *Bombus terrestris* (Hymenoptera: Apidae). *Gene*, **390**, 52-66.
6. Keravala, A., Liu, D., Lechman, E.R., Wolfe, D., Nash, J.A., Lampe, D.J. & Robbins PD. (2006) Hyperactive *Himar1* transposase mediates transposition in cell culture and enhances gene expression *in vivo*. *Hum. Gene Ther.* **17**, 1006-1018.
7. Wilber, A., Frandsen, J.L., Geurts, J.L., Largaespada, D.A., Hackett, P.B., & McIvar, R.S. (2006) RNA as a source of transposase for Sleeping Beauty-mediated gene insertion and expression in somatic cells and tissues. *Mol. Ther.* **13**, 625-630
8. Zuker, M. (2003) Mfold web server for nucleic acid folding and hybridization prediction. *Nucl. Acids Res.* **31**, 3406-3415.
9. Augé-Gouillou, C., Hamelin, M.H., Demattei MV, Periquet G, & Bigot, Y. (2001) The ITR binding domain of the Mariner Mos-1 transposase. *Mol. Genet. Gen.* **265**, 58-65.
10. Wautot, V., Khodaei, S., Frappart, L., Buisson, N., Baro, E., Lenoir, G.M., Calender, A., Zhang, C.X. & Weber, G. (2006) Expression analysis of endogenous menin, the product of the multiple endocrine neoplasia type 1 gene, in cell lines and human tissues. *Int. J. Cancer*, **85**, 877-881.
