# Supplementary material for: Nuclear Importation of Mariner Transposases among Eukaryotes: Motif Requirements and Homo-Protein Interactions
Source: PLoS One. 2011 Aug 18;6(8):e23693. doi: 10.1371/journal.pone.0023693 (PMC3158080; doi:10.1371/journal.pone.0023693)
Supplement: Supporting Information S6 — Oligonucleotides used in the studies. (PDF) [file pone.0023693.s006.pdf]

Nuclear Importation of *Mariner* Transposases among Eukaryotes: Motif Requirements, and Homo-Protein Interactions

Marie-Véronique Demattei, Sabah Hedhili, Ludivine Sinzelle, Christophe Bressac, Sophie Casteret, Nathalie Moiré, Jeanne Cambefort, Xavier Thomas, Nicolas Pollet, Pascal Gantet and Yves Bigot

Supporting Information 6 : Oligonucleotides used in the studies

Primer name	Nucleotide sequence	Positions in Tpase sequence*
	Mos1	
Mos1-5'NcoI	5'-GCGTCACCATGGCGAGTTTCGTGCCG-3'	1-6
Mos1-3'SpeI	5'-GACTAGTTTCAAAGTATTTGCCGTCGCT-3'	345-339
Mos1-3'Δ1 SpeI :	5'-GACTAGTAGCATCGTCTTCATCCAATAA-3'	86-79
Mos1-3'Δ2 SpeI:	5'-GACTAGTTCCAGGATCAACGTATGACT-3'	175-169
Mos1-3'Δ3 NcoII	5'-CCCATGGGACAACCGTCCACATCG-3'	175-180
Mos1-Δ4 NcoI	5'-GCGTCACCATGGAAACGCAAAAACAACTCG-3'	86-93
Mos1-Δ5 NcoI	5'-GCGTCACCGAAGTAAGTCAACAAGCAGTTT-3'	97-104
Mos1-Δ6 NcoI	5'-GCGTCACCGTGATCTTCCTGCACGACAATG-3'	244-251
Mos1-NLS1up	5'-CATGGAACGCTTCAAAAGTGGTGATTTTGACGTCGACGACAAAGA GCACGGAAAACCGCCAAAAAGGA-3'	52-71
Mos1-NLS1dw	5'-CTAGTCCTTTTTGGCGGTTTTCCGTGCTCTTTGTCGTCGACGTCA AAATCACCACTTTTGAAGCGTTC-3'	52-71
Mos1-NLS2up	5'-CATGGGAAAACCGCCAAAAAGGTACGA-3'	66-71
Mos1-NLS2dw	5'-CTAGCGTACCTTTTTGGCGGTTTTCC-3'	66-71
Mos1-NLS3up	5'-CATGGAGAGGCGCAAAAACACATGCGAAATTTTGCTTTCACGATA CAAAAGGA-3'	131-146
Mos1-NLS3dw	5'-CTAGTCCTTTTGTATCGTGAAAGCAAAATTTCGCATGTGTTTTTG CGCCTCTC-3'	131-146
Mos1-NLS4up	5'-CATGCCTAAACGTAAAAAGTCATACG-3'	164-169
Mos1-NLS4dw	5'-CTAGCGTATGACTTTTTACGTTTAGG-3'	164-169
	Himar1	
Himar1-5'NcoI	5'-GCCATGGAAAAAAAGGAATTTCG-3'	1-7
Himar1-3'SpeI	5'-GACTAGTTTCAACATAGTTCCCTTCAAG-3'	348-342
Himar1-3'Δ1SpeI	5'-GACTAGTATTCAAAATCATTTGTGGAT-3'	82-76
Himar1-3'Δ2SpeI	5'-GACTAGTGGTCGCTGTCCACTCAGCCGA-3'	179-173
Himar1-3'Δ3NcoI	5'-CCCATGGCTGAGTGGACAGCGACC-3'	174-179
	Mcmar1	
Mcmar1-5'NcoI	5'-GCCATGGATGAAAAAAAAAGAATAAG-3'	1-8
Mcmar1-3'SpeI	5'-GACTAGTTTCATAGAAATAATTTCCATT-3'	340-334
Mcmar1-3'Δ1SpeI	5'-GACTAGTTGCGGGATTCTCATTAATTGC-3'	82-76
Mcmar1-3'Δ2SpeI	5'-GACTAGTCCCAACTGCAAGCCATTGCTT-3'	171-165
Mcmar1-3'Δ3NcoI	5'-CCCATGCAAGATGCTAATGCAA-3'	172-176

* in the alignment of Fig. 1
